# Natural killer cells, gamma delta T cells and classical monocytes are associated with systolic blood pressure in the multi-ethnic study of atherosclerosis (MESA)

**DOI:** 10.1186/s12872-021-01857-2

**Published:** 2021-01-22

**Authors:** Joseph A. C. Delaney, Nels C. Olson, Colleen M. Sitlani, Alison E. Fohner, Sally A. Huber, Alan L. Landay, Susan R. Heckbert, Russell P. Tracy, Bruce M. Psaty, Matt Feinstein, Margaret F. Doyle

**Affiliations:** 1 College of Pharmacy, University of Manitoba, Winnipeg, MB Canada; 2 Department of Epidemiology, University of Washington, Seattle, WA USA; 3 Department of Pathology and Laboratory Medicine, Larner College of Medicine, University of Vermont, Burlington, VT USA; 4 Department of Medicine, University of Washington, Seattle, WA USA; 5 Division of Geriatrics, Gerontology and Palliative Medicine, Department of Internal Medicine, Rush University Medical Center, Chicago, IL USA; 6 Department of Biochemistry, Larner College of Medicine, University of Vermont, Burlington, VT USA; 7 Cardiovascular Health Research Unit, Departments of Medicine, Epidemiology, and Health Services, University of Washington, Seattle, WA USA; 8 Kaiser Permanente Washington Health Research Institute, Seattle, WA USA; 9 Division of Cardiology, Department of Medicine, Northwestern University Feinberg School of Medicine, Chicago, IL USA

**Keywords:** Lymphocytes, Innate immunity, Adaptive immunity, Systolic blood pressure, Cryopreserved cells, Longitudinal cohort study, Γδ T cells, Monocytes

## Abstract

**Background:**

Hypertension is a major source of cardiovascular morbidity and mortality. Recent evidence from mouse models, genetic, and cross-sectional human studies suggest increased proportions of selected immune cell subsets may be associated with levels of systolic blood pressure (SBP).

**Methods:**

We assayed immune cells from cryopreserved samples collected at the baseline examination (2000–2002) from 1195 participants from the multi-ethnic study of atherosclerosis (MESA). We used linear mixed models, with adjustment for age, sex, race/ethnicity, smoking, exercise, body mass index, education, diabetes, and cytomegalovirus titers, to estimate the associations between 30 immune cell subsets (4 of which were a priori hypotheses) and repeated measures of SBP (baseline and up to four follow-up measures) over 10 years. The analysis provides estimates of the association with blood pressure level.

**Results:**

The mean age of the MESA participants at baseline was 64 ± 10 years and 53% were male. A one standard deviation (1-SD) increment in the proportion of γδ T cells was associated with 2.40 mmHg [95% confidence interval (CI) 1.34–3.42] higher average systolic blood pressure; and for natural killer cells, a 1-SD increment was associated with 1.88 mmHg (95% CI 0.82–2.94) higher average level of systolic blood pressure. A 1-SD increment in classical monocytes (CD14^++^CD16^−^) was associated with 2.01 mmHG (95% CI 0.79–3.24) lower average systolic blood pressure. There were no associations of CD4^+^ T helper cell subsets with average systolic blood pressure.

**Conclusion:**

These findings suggest that the innate immune system plays a role in levels of SBP whereas there were no associations with adaptive immune cells.

## Background

Hypertension is a major source of cardiovascular morbidity and mortality in the United States [1, 2]. Major risk factors for hypertension include dietary sodium, physical inactivity, alcohol intake, and obesity [1]. While effective dietary and drug therapies exist to treat elevated blood pressure, our understanding of the biology and causes of hypertension remains incomplete.

Human and animal studies suggest that both the innate and adaptive immune system may be related to hypertension [3–7] although samples sizes have been limited. This link between immune cells and hypertension may help explain previous associations seen between immune cells and atherosclerosis. In a previous cross-sectional publication from the Multi-Ethnic Study of Atherosclerosis (MESA) Exam 4 (2005–2007), fresh samples were assayed for lymphocyte subsets. Investigators found that naive and memory CD4^+^ T cells, and T helper type 1 (Th1) cells, analyzed as proportions of CD4^+^ cells, were associated with subclinical atherosclerosis [8, 9]. These fresh lymphocytes were from a subset of participants, at a later exam, and fewer white-cell subsets were measured in this prior study.

To investigate the links between immune cell subsets and cardiovascular disease (CVD), we used cryopreserved peripheral blood mononuclear cells (PBMC) to phenotype a range of immune cells by flow cytometry in the MESA cohort [10]. We leveraged the availability of this data for a secondary analysis of the relationships between 30 immune cell subsets and repeated SBP measures measured over 10-years. Based on prior findings [3–7] that some immune cell subsets were associated with blood pressure (primarily in mouse models), we hypothesized a priori that higher proportions of Th1, gamma delta (γδ) T cells, and Th17 cells would be associated with higher systolic blood pressure, and that higher proportions of regulatory T cells would be associated with lower systolic blood pressure [3, 7, 11, 12]. We included the other 26 immune cell subsets measured in MESA as secondary hypotheses, to understand broadly the relationship between the immune system and systolic blood pressure.

## Methods

### Study design

MESA is a community-based sample of 6814 men and women aged 45–84 years and recruited from 6 field centers (Baltimore, MD; Chicago, IL; Forsyth County, NC; Los Angeles County, CA; northern Manhattan, NY; and St. Paul, MN). Participants self-identified as White, Black, Hispanic, or Chinese. Exclusion criteria for the MESA cohort included a self-reported medical history of myocardial infarction, angina, cardiovascular procedures, heart failure, cerebrovascular disease, active treatment for cancer, pregnancy or amputation. Details regarding design, recruitment, and objectives of MESA are described elsewhere [13]. We designed a case-cohort study to test the primary hypothesis that immune cells subsets were risk factors for CHD [10]. We then leveraged the data from the case-cohort study to consider other scientific questions using the same study population [14]. We selected a case-cohort sample of 1195 participants, with case status (n = 476) based on incident myocardial infarction or incident angina events during study follow-up [10] with the remainder of the participants being selected by simple random sampling.

The original case cohort study of incident MI/angina was designed from the main MESA baseline cohort using cryopreserved cells stored at baseline (2000–2002) [10]. The original selection, n = 1200, was determined based on statistical power for the primary study [10]. Five of the selected participants had no sample in the repository. For the remaining 1195 participants, each sample was split into 6 assay panels as described in Additional file 2: Table S1. The number of cellular phenotypes measured per participant varied due to occasional poor sample quality of the individual PBMCs or technical errors. This was particularly true in the Th1, Th2, Th17 samples where a key reagent was incorrectly prepared for a portion of the participants, resulting in unreportable results. After finalizing the flow cytometry data, the number of participants with valid phenotypes varied from 770 to 1113. Since the sample quality and technical errors were assumed to be unrelated to participant characteristics in post-hoc analysis, we treated this data as missing at random.

Therefore, using weights derived from the case-cohort sampling to account for over-sampling of events, we included all participants with available data from this case-cohort study to investigate the associations of immune cell subsets with measures of systolic blood pressure over the course of the study. To be specific, we used MESA data from Exams 1–5 over 10 years of study follow-up, all of which included systolic blood pressure measures.

University of Washington IRB Committee J reviewed the MESA cohort study and approved it as FWA #00006878.

### Data collection

At the baseline (2000–2002) and follow-up clinical visits (in 2002–2004, 2004–2005, 2005–2007, and 2010–2012) standardized questionnaires and calibrated devices were utilized to obtain data on demographics, tobacco use, medical conditions, prescription medication usage, weight, and height. PBMCs, used in this study, were collected at baseline. At each MESA exam, resting, seated blood pressure was measured 3 times using a Dinamap automated oscillometric sphygmomanometer (model Pro 100; Critikon, Tampa, Florida); the last 2 measurements were averaged for analysis. To adjust for hypertension treatment (33% of MESA participants were treated for hypertension at baseline), we added 10 mmHg to systolic blood pressure for treated participants, based on an approach that is widely used in genome-wide association studies (GWAS) [15, 16], although sometimes higher values are proposed for this correction [17].

### Immune cell subsets

Detailed methods for immune cell phenotyping and flow cytometry gating strategies have been published [10] and specific antibodies utilized are shown in Additional file 2: Table S1. Briefly, as part of the baseline MESA exam, an 8 mL citrate CPT tube was drawn, PBMCs were collected, washed and frozen in freezing media containing 90% fetal bovine serum (FBS), 10% dimethyl sufoxide (DMSO) utilizing a controlled freezing rate. Cells (2 × 1 mL aliquots) were stored in a − 135 °C freezer until thawed. Cells (1 mL aliquot) were placed in a 37 °C water bath for 15 min until thawed. Cells were slowly diluted by addition of media with gentle mixing. For intracellular staining of CD4^+^ (Th1, Th2, Th17) and CD8^+^ (Tc1, Tc2, Tc17), cells were activated for 3 h at 37 °C with phorbal myristic acetate & ionomycin, and stained with live/dead stain. Cells were then surface labeled for CD4/CD8, fixed with paraformaldehyde and intracellularly stained for interferon gamma (IFN-γ), interleukin 4 (IL-4), and interleukin-17A (IL-17A) [10]. All other cellular phenotypes were surface labeled with antibodies as shown in Additional file 2: Table S1 and previously described [10]. Briefly, samples were centrifuged and placed into phosphate buffer saline at pH 7.4, then labeled with live/dead stain for 20 min. The stain was removed by centrifugation; cells were resuspended and labeled with specified cell surface markers for 20 min. Cells were washed, and fixed in paraformaldehyde, and stored in the dark at 4 °C prior to analysis on a MACSQuant 10 flow cytometer (Miltenyi Biotec, Germany), calibrated daily. Single color controls were used for compensation and isotype controls were used for negative gate setting. Results were analyzed using MacsQuantify software (Miltenyi Biotec). Gating strategies were previously reported [10].

### Statistical analysis

Table 2 shows the immune cells analyzed, the markers used to identify these cells, the parent population, the number of valid measurements for each cell type and their means/standard deviations. All data is presented as a percent of the given parent population, which we treated as continuous variables for the purpose of analysis. Associations of immune cell subsets with systolic blood pressure were estimated relative to a one standard deviation (1-SD) difference in each of the cell proportions.

We used linear mixed models to evaluate the associations between immune cell subsets measured at MESA baseline and systolic blood pressure levels over 10 years, across five MESA exams. Models were adjusted for covariates measured at baseline that were thought to be possible confounders, including age, sex, race/ethnicity, smoking, exercise, body mass index (BMI), college education, diabetes, and log transformed cytomegalovirus antibody titer [8, 9]. Categorical variables with more than two levels were converted to indicator variables, two level categorical variables were included as indicator variables, and continuous variables included as linear adjustment variables, except for cytomegalovirus antibody titer which was log-transformed for skew. Immune-subset exposures were modeled singly, to avoid collinearity between the subsets and to maximize sample size, given that rates of missingness varied (Table 2).

These mixed models handled within-person correlation due to each subject having up to five systolic blood pressure measurements with subject-specific random intercepts. Inverse probability of sampling weights were used to account for the case-cohort sampling scheme. Confidence intervals used robust (sandwich) standard error estimates to ensure validity in the presence of the sampling weights. We examined the residuals of the linear regression model to determine whether the residuals were normally distributed and thus estimation using linear mixed models was appropriate. We used Bonferroni correction as our primary approach for controlling for multiple testing [18], despite the known correlations between the different immune cell subsets, some of which were high (making it overly conservative). The Bonferroni corrected *p* value threshold was 0.05/4 = 0.0125 for our primary hypotheses, and for our secondary hypotheses was 0.05/30 = 0.0017. Additionally, a false-discovery rate (FDR) approach [19] was implemented in a sensitivity analysis as an alternative to the overly conservative Bonferroni approach, which assumes independence of measures.

**Fig. 1:**
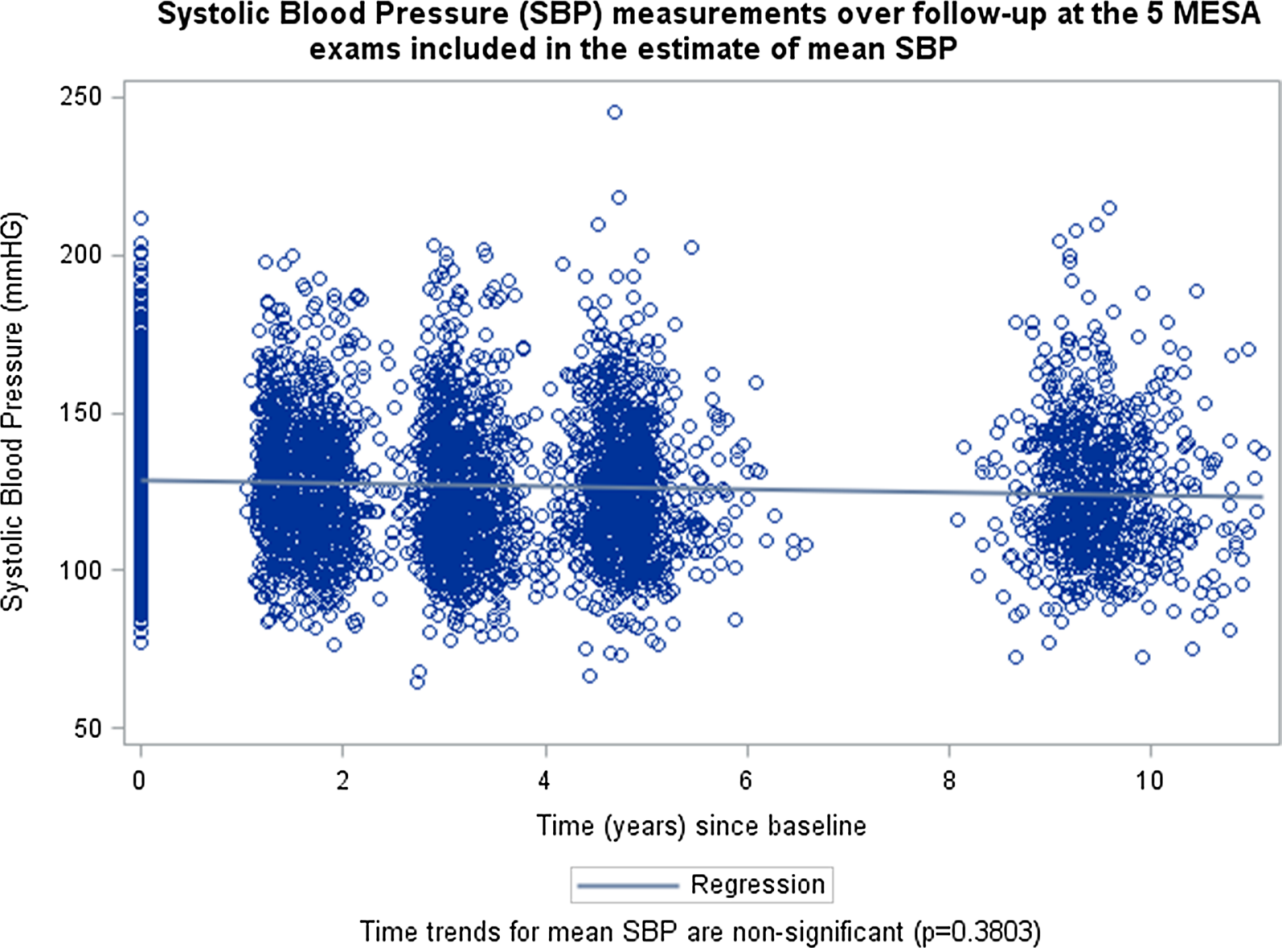
Systolic blood pressure (SBP) measurements over follow-up at the 5 MESA exams included in the estimate of mean SBP

Primary analyses used all available data for each immune cell subset, under the assumption that missing data were missing completely at random. We included the SBP value at each of the five MESA exams as a repeated measure of blood pressure and thus estimates of association are with the average level of blood pressure, not with changes in blood pressure over time. Mixed models were used to handle irregular data sampling, differences in timing between measures, and the same participant contributing between one and five measures. Figure 1 shows the unadjusted blood pressure, with measures clustering around each of the follow-up time points (participant baseline is time zero by MESA study design). We tested for time trends, to ensure that we did not need to account for them in our model.

We performed exploratory analysis for immune cell interactions by age, sex, race, and BMI for the primary hypotheses and any secondary hypothesis that was deemed significant after multiple testing correction. We tested for effect measure modification by antihypertensive medication use, using medication exposure as a time-varying covariate.

## Results

The case-cohort study sample included 1195 MESA participants, with a mean (SD) age of 64 (± 10) years, and 53% of whom were male. The mean (SD) baseline systolic blood pressure was 130.0 mmHG (21.7 mmHg) at the Exam 1 baseline visit (Table 1). The average blood pressure at baseline and the subsequent follow-up exams can be seen in Fig. 1, and it does not show a statistically significant trend with time over study follow-up.

**Table 1:** Characteristics of MESA subjects (n = 1087) with measures for γδ T cells, as a representative of a typical population in the study*

Variable	Mean/number	Standard Deviation (SD)/percent
Age (years, SD)	63.9	10.1
Male (n, %)	578	53%
Race/ethnicity (n, %)		
White	413	38%
Chinese	135	12%
Black	314	29%
Hispanic	227	21%
Diabetes (n, %)	184	17%
Smoker (n, %)	144	13%
Alcohol user (n, %)	557	64%
Statin user (n, %)	191	18%
Calcium Channel Blocker user (n, %)	159	15%
Diuretic user (n, %)	173	16%
Beta Blocker user (n, %)	110	10%
Vasodilator user (n, %)	67	6%
ACE User (n, %)	171	16%
College education (n, %)	378	35%
Intentional Exercise (met-min/week, SD)	1527	2293
IL-6 (pg/mL, SD)	1.64	1.24
CMV antibody titer (EU/mL, SD)	240	266
Systolic blood pressure (mmHG, SD)	130.0	21.7
Diastolic blood pressure (mmHG, SD)	72.7	10.4
Total cholesterol (mg/dL, SD)	193.9	35.8
HDL Cholesterol (mg/dL, SD)	49.4	14.4
Body Mass Index (kg/m^2^, SD)	28.3	5.4

All values were taken from participants at study baseline

The proportions of immune cell subsets and the number of participants contributing data to each are presented in Table 2. For example, γδ T cells averaged 6.6% of the total CD3^+^ cells measured. The numbers of participants with data on any particular immune cell phenotype ranged from 770 to 1113.

**Table 2:** Cellular phenotypes with their molecular description, parent population, number of samples evaluated, means and standard deviations

Cellular phenotype	Molecular description	Parent population	N	Mean	SD
Primary hypotheses					
Th1	CD4^+^ IFN-γ^+^	CD4^+^	770	15.3	9.0
Th17	CD4^+^ IL-17A^+^	CD4^+^	770	2.1	1.4
Tregs	CD4^+^ CD25^+^ CD127^−^	CD4^+^	1113	6.2	3.8
γδ T cells	CD3^+^ γδ^+^	CD3^+^	1087	6.6	6.1
Exploratory hypotheses					
T cells	CD3^+^	% Lymphocytes	1087	62.7	13.7
B cells	CD19^+^	% Lymphocytes	1087	11.3	7.4
NK cells	CD3^−^ CD56^+^ CD16^+^	% Lymphocytes	1087	5.0	5.7
Classical monocytes	CD14^++^ CD16^−^	CD14 total	922	74.4	10.2
Intermediate monocytes	CD14^+^ CD16^+^	CD14 total	922	18.1	7.1
Non-classical monocytes	CD14_Dim_CD16^+^	CD14 total	922	7.4	7.5
T helper cells	CD4^+^	% Lymphocytes	1051	50.0	11.0
Th2 cells	CD4^+^ IL-4^+^	CD4^+^	770	2.9	1.8
Naive CD4^+^ cells	CD4^+^ CD45RA^+^	CD4^+^	1051	26.2	12.1
Memory CD4^+^ cells	CD4^+^ CD45RO^+^	CD4^+^	1051	51.7	13.4
CD28-senescent CD4 cells	CD4^+^ CD28^−^	CD4^+^	1051	13.9	10.0
CD28-CD57^+^ senescent CD4 cells	CD4^+^ CD28^−^ CD57^+^	CD4^+^	1051	9.9	8.5
CD4^+^ T_EMRA_	CD4^+^ CD45RA^+^ CD28^−^ CD57^+^	CD4^+^	1051	5.6	5.3
Activated/mature CD4^+^ cells	CD4^+^ CD38^+^	CD4^+^	1051	26.1	12.1
CD57^+^ CD4^+^ cells	CD4^+^ CD57^+^	CD4^+^	1051	22.4	13.0
Cytotoxic T cells	CD8^+^	% Lymphocytes	1062	23.6	9.3
Tc1	CD8^+^ IFN-γ^+^	CD8^+^	770	41.8	17.9
Tc2	CD8^+^ IL-4^+^	CD8^+^	770	7.1	4.9
Tc17	CD8^+^ IL-17A^+^	CD8^+^	770	5.4	5.8
Naive CD8^+^ cells	CD8^+^ CD45RA^+^	CD8^+^	1062	52.4	14.7
Memory CD8^+^ cells	CD8^+^ CD45RO^+^	CD8^+^	1062	21.7	10.6
CD28-senescent CD8 cells	CD8^+^ CD28^−^	CD8^+^	1062	55.6	15.9
CD28^−^ CD57^+^ senescent CD8 cells	CD8^+^ CD28^−^ CD57^+^	CD8^+^	1062	44.5	15.9
CD8^+^ T_EMRA_	CD8^+^ CD45RA^+^ CD28^−^ CD57^+^	CD8^+^	1062	32.8	14.3
Activated/mature CD8^+^ cells	CD8^+^ CD38^+^	CD8^+^	1062	23.6	12.2
CD57^+^ CD8^+^ cells	CD8^+^ CD57^+^	CD8^+^	1062	59.3	15.4

Of the four primary immune cell subsets that comprised our a priori hypotheses group (γδ T, Th1 (CD4^+^IFN-γ^+^), Th17 (CD4^+^IL-17A^+^), and Tregs (CD4^+^CD25^+^CD127^−^)), only γδ T cells were significantly associated with systolic blood pressure (Table 3). A one standard deviation (1-SD) increment in the proportion of γδ T cells was associated with a 2.40 mmHg [95% confidence interval (CI) 1.34–3.42] higher level of systolic blood pressure. This association was significant after Bonferroni correction.

**Table 3:** Associations between lymphocyte subsets (per 1-SD) and average systolic blood pressure level (mmHG) across 10 years of follow-up

	∆ mmHG (per SD)	95% CI lower	95% CI upper	*p *value
Primary hypotheses (significance threshold *p* < 0.0125)				
Th1	1.19	− 0.41	2.79	0.15
Th17	− 0.06	− 1.31	1.18	0.92
Tregs	1.09	0.05	2.13	0.04
γδ T	2.40	1.34	3.42	< 0.0001
Exploratory hypotheses (significance threshold *p* < 0.0017)				
T cells	− 1.22	− 2.34	− 0.09	0.03
B cells	− 0.45	− 1.6	0.7	0.44
NK cells	1.88	0.82	2.94	0.0005
Classical monocytes	− 2.01	− 3.24	− 0.79	0.0013
Intermediate monocytes	0.98	− 0.31	2.28	0.14
Non-classical monocytes	1.82	0.64	3.00	0.0025
CD4^+^	− 0.15	− 1.19	0.89	0.78
Th2	− 0.14	− 1.45	1.18	0.84
Naïve CD4^+^	0.18	− 0.97	1.34	0.76
Memory CD4^+^	0.49	− 0.68	1.65	0.41
CD4^+^ CD28^−^	− 0.19	− 1.34	0.96	0.75
CD4^+^ CD28^−^ CD57^+^	− 0.04	− 1.17	1.08	0.94
CD4^+^ T_EMRA_	0.45	− 0.53	1.43	0.37
CD4^+^ CD38^+^	− 0.36	− 1.51	0.80	0.54
CD4^+^ CD57^+^	0.05	− 1.07	1.17	0.93
CD8^+^	0.11	− 1.02	1.24	0.85
Tc1	1.24	− 0.11	2.59	0.07
Tc2	− 0.93	− 2.21	0.34	0.15
Tc17	0.49	− 0.90	1.88	0.49
Naïve CD8^+^	0.97	− 0.15	2.09	0.09
Memory CD8^+^	− 1.02	− 2.13	0.09	0.07
CD8^+^ CD28^−^	0.91	− 0.28	2.09	0.13
CD8^+^ CD28^−^ CD57^+^	0.69	− 0.46	1.84	0.24
CD8^+^ T_EMRA_	0.99	− 0.13	2.12	0.08
CD8^+^ CD38^+^	0.53	− 0.58	1.64	0.35
CD8^+^ CD57^+^	0.42	− 0.67	1.52	0.45

Statistical models are linear mixed models using repeated blood pressure measures for MESA exams 1–5, and adjusted for baseline age, sex, race/ethnicity, smoking, exercise, body mass index, college education, diabetes, and log transformed CMV antibody titer

After adjusting for multiple comparisons, three other immune cell subsets, included in our exploratory secondary analyses, were also associated with systolic blood pressure; two were significant using a Bonferroni criteria (natural killer cells and classical monocytes), while using FDR added one additional immune cell subset (non-classical monocytes). A 1-SD increment in the proportion of natural killer cells was associated with a 1.88 mmHG (95% CI 0.82–2.94) higher level of systolic blood pressure during follow-up. The two other immune cell subsets associated with systolic blood pressure were both monocytes. A 1-SD increase in classical monocytes (characterized as CD14^++^CD16^−^) was associated with a 2.01 mmHG (95% CI 0.79–3.24) lower level of systolic blood pressure. While barely missing the Bonferroni threshold, a 1-SD increment in non-classical monocytes (characterized as CD14_dim_CD16^++^) was associated with a 1.82 mmHG (95% CI 0.64–3.00) higher level of systolic blood pressure. In sensitivity analyses, using a false discovery rate approach [19] instead of the Bonferroni approach (FDR cutoff for non-classical monocytes is 0.0068 versus an observed p-value of 0.0025), results for non-classical monocytes would also be considered statistically significant.

In further exploratory analyses, we looked for interaction of our main findings with sex, race, BMI, and age. The data, shown in the Additional file 2: Tables S2–S5, were null for all interactions, although some interactions had relatively large point estimates that were imprecise and could not exclude the null hypothesis. There was some limited evidence of effect modification for γδ T cells by the use of antihypertensive medications (*p* = 0.0203) (Table 4). For all of the other cell types, there was no statistically significant evidence of effect measure modification on the linear scale.

**Table 4:** Stratification by anti-hypertensive medication use of the immune cell subsets with a statistically significant main effect to test for effect measure modification

Immune cell subset	∆ mmHG (per SD)	95% CI		*p* value
Any antihypertensive medication use (median SBP 131 mmHG) n = 1852 repeated SBP measures				
CD3^+^ γδ^+^	5.10	3.03	7.17	< .0001
CD3^−^ CD56^+^ CD16^+^	2.25	1.06	3.45	0.0002
CD14^++^ CD16^−^	− 2.02	− 3.61	− 0.43	0.0127
CD14^+^ CD16^+^	1.16	− 0.41	2.74	0.1475
CD14_Dim_CD16^++^	1.52	0.019	3.30	0.0472
No antihypertensive medication use (median SBP 119.5 mmHG) n = 1928 repeated SBP measures				
CD3^+^ γδ^+^	1.33	0.22	2.45	0.0194
CD3^−^ CD56^+^ CD16^+^	0.76	− 0.67	2.19	0.2997
CD14^++^ CD16^−^	− 0.79	− 2.22	0.63	0.2756
CD14^+^ CD16^+^	− 0.38	− 1.76	1.00	0.5912
CD14_Dim_CD16^++^	1.56	− 0.027	3.15	0.0540

Numbers refer to blood pressure readings under treatment, as each participant may contribute multiple measures and may change antihypertensive medication exposure category over follow-up. ∆ mmHG (per SD) refers to a change in the average level of SBP over 10 years of follow-up

## Discussion

The main finding of this study is the association of γδ T cells, natural killer cells, and two monocyte populations with average systolic blood pressure during 10 years of measurement in a large multi-ethnic population. Three of these immune-cell subsets-γδ T cells, natural killer cells, and non-classical monocytes-are pro-inflammatory cells, which may suggest an association between innate immune cell-mediated inflammation and systolic blood pressure in a multi-ethnic cohort with no baseline cardiovascular disease.

One of our a priori hypotheses, the association between higher proportions of γδ T cells and higher systolic blood pressure replicates the association seen in Caillon et al. [3] and further builds evidence that these cells are involved in the development of human hypertension. γδ T cells respond rapidly in the initiation phase of immune reactions and act as a “bridge” between the innate and adaptive systems [20]. The current data are consistent with animal models of hypertension where γδ T cell receptor gene deletion or addition of inhibitory γδ T cell receptor antibodies blunted endothelial dysfunction and hypertension in an angiotensin II model of hypertension in mice [3]. γδ T cells produce the cytokine IL-17 that has been implicated in hypertension [6, 21–25]. When antibodies to IL-17 were administered in a mouse model of hypertension, hypertension was attenuated, renal and vascular cellular infiltration and proinflammatory proteins, such as TGF-β, were decreased [12, 24]. In the current study, we did not see an association of the IL-17 producing Th17 cells with systolic blood pressure, indicating the results seen previously indicating a role for IL17 may have been due to IL-17 production by γδ T cells or other IL17-producing cells.

In our exploratory analysis, we discovered an association between increased proportions of natural killer cells and levels of systolic blood pressure. Like γδ T cells, NK cells play a critical role in viral infection [20]. Natural killer cells are non-specific responders to bacterial or viral particles or infected cells and can produce IFN-γ and other cytokines that have been shown to be associated with hypertension [6, 21–26]. IFN-γ knockout mice were protected from angiotensin II induced vascular and kidney dysfunction [23]. In contrast, IFN-γ receptor knock out mice did not have the response to angiotensin II induced hypertension, although cardio-protective effects were noted [27]. While the current study does not show an association with the IFN-g producing adaptive immune cells (Th1, Tc1), the association of NK cells, which are a major source of IFN-γ, supports the role of IFN-γ in hypertension in humans.

Monocytes are an important component of the innate immune system and previous studies have implicated monocytes in atherosclerosis [28]. Monocytes have shown associations with blood pressure in several animal models [7, 29, 30] and these cells are related to both tissue remodeling in the vasculature as well as vascular inflammation [7]. Current anti-hypertensive medications (such as Angiotensin-converting enzyme inhibitors and Angiotensin II Receptor Blockers) can directly influence monocyte behavior, making it a plausible target for therapy [7]. The associations with systolic blood pressure observed here, with a shift from classical to the more pro-inflammatory non-classical monocytes, suggest a potential for further refinement of possible drug targets as well as a better understanding of the origins of hypertension. Notably, this may be an especially attractive line of research as monocytes are also thought to be involved in organ damage due to hypertension [7].

These data support other recent studies showing important links between the immune system and diseases, including both cardiovascular and kidney disease [31–33]. This includes evidence that the neutrophil-to-lymphocyte ratio is a predictor of mortality and/or kidney dysfunction in older patients with hypertension [31, 32], although neither of these studies looked at antihypertensive medications. This makes the possible effect modification of γδ T cells with anti-hypertensive medication use observed in the current study of potential interest for designing prospective future studies with adequate power linking the immune system and hypertension for prediction. Finally, there is a clear advantage in being able to sub-type immune cells, as cruder approaches to classifying immune cells do not show significant differences between hypertensive and non-hypertensive participants [25].

The strengths of this study include the large sample size, the large panel of cell subsets evaluated and the long term, longitudinal measures of systolic blood pressure. The limitations include the observational nature of the data and technical issues with complex samples which resulted in some missing data. The use of cryopreserved PBMCs may result in different absolute levels of some of the subsets as compared with whole blood [25], although relative levels should be preserved [34]. Further, the immune cell distributions measured in this study from cryopreserved samples are similar to those previously measured in fresh whole blood obtained in MESA at Exam 4 (2005–2007) [8, 9] among phenotypes measured in both studies. Furthermore, participants treated with different anti-hypertensive medications may alter underlying biological relationships between some immune cell subsets and systolic blood pressure. Due to the variety of medications used in this cohort, we were not powered to see these relationships. It is also unknown how interventions on the immune cells will translate into therapeutic results; additional intervention studies will be required to allow translation of these results [35]. As a general correction for the direct effect of the medication, we added 10 mmHg to participants’ systolic blood pressure who were being treated [15–18], and any approach to accounting for medication use can have some measurement error.

## Conclusions

In conclusion, this study provides evidence that γδ T cells, natural killer cells and classical and non-classical monocytes are associated with systolic blood pressure levels in a large multi-ethnic cohort. These data support previous animal studies that show a role for innate immune cells in affecting systolic blood pressure levels, and the consequent possibility of hypertension.

## Supplementary Information

**Additional file 1**. Sample SAS code used to build the dataset used in this analysis and the exact statistical models used.

**Additional file 2**. Supplmentary tables.

**Additional file 3**. Sample MESA informed consent form.

## Data Availability

The full MESA dataset can be requested from the Collaborative Health Studies Coordinating Center (CHSCC) upon approval of a paper proposal using this data. The instructions are at https://www.mesa-nhlbi.org/. MESA data is also available via BIOLINCC (https://biolincc.nhlbi.nih.gov/home/). The full code used to build the data sets and analyze the primary analysis is included as an Additional file 1 to this paper. The instruments and questionnaires given to the subjects are available at https://www.mesa-nhlbi.org/ex1forms.aspx#exam.

## Abbreviations

1-SD: One standard deviation
BMI: Body mass index
FDR: False-discovery rate
MESA: Multi-ethnic study of atherosclerosis
PBMC: Peripheral blood mononuclear cells
SBP: Systolic blood pressure
Th1: T helper type 1
Th2: T helper type 2
Th17: T helper type 17
