# Supplementary material for: Natural killer cells, gamma delta T cells and classical monocytes are associated with systolic blood pressure in the multi-ethnic study of atherosclerosis (MESA)
Source: BMC Cardiovasc Disord. 2021 Jan 22;21:45. doi: 10.1186/s12872-021-01857-2 (PMC7821496; doi:10.1186/s12872-021-01857-2)
Supplement: Supplementary file 1 — Additional file 1. Sample SAS code used to build the dataset used in this analysis and the exact statistical models used. [file 12872_2021_1857_MOESM1_ESM.pdf]

```
** This Program is the data assembly and analysis code for the
Lymphocytes and Systolic Blood Pressure Paper as of December 2019 **;


/* First, assemble original lymphocyte data */

libname lib 'U:\T Cell Project\Data';
libname LIB2 'U:\T Cell Project\Nels\';

data quat;
set lib2.colleen_enhanced (keep=idno samp_weight);
run;


data check_1;
set lib.MESA_Data_export_20170120;

idno=mesaid;
if LIVE_LYMPH_COUNT < 0 then LIVE_LYMPH_COUNT =.;
if LIVE_LYMPH_COUNT_8 < 0 then LIVE_LYMPH_COUNT_8 =.;
if  cd4_mnas_mesa_final_CD4_    < 0 then    cd4_mnas_mesa_final_CD4_    =. ;
if  CD4_CD45RA_ < 0 then    CD4_CD45RA_ =. ;
if  CD4_CD45RO_ < 0 then    CD4_CD45RO_ =. ;
if  CD4_CD28_CD57_  < 0 then    CD4_CD28_CD57_  =. ;
if  CD4_CD38_   < 0 then    CD4_CD38_   =. ;
if  CD4_CD28_   < 0 then    CD4_CD28_   =. ;
if  CD4_CD45RA_CD28_CD57__AS_CD4    < 0 then    CD4_CD45RA_CD28_CD57__AS_CD4    =. ;
if  CD4_CD57_   < 0 then    CD4_CD57_   =. ;
if  cd8_mnas_mesa_final_CD8_    < 0 then    cd8_mnas_mesa_final_CD8_    =. ;
if  CD8_CD45RA_ < 0 then    CD8_CD45RA_ =. ;
if  CD8_CD45RO_ < 0 then    CD8_CD45RO_ =. ;
if  CD8_CD28_CD57_  < 0 then    CD8_CD28_CD57_  =. ;
if  CD8_CD38_   < 0 then    CD8_CD38_   =. ;
if  CD8_CD28_   < 0 then    CD8_CD28_   =. ;
if  CD8_CD45RA_CD28_CD57__AS_CD8    < 0 then    CD8_CD45RA_CD28_CD57__AS_CD8    =. ;
if  CD8_CD57_   < 0 then    CD8_CD57_   =. ;
if  cd3_    < 0 then    cd3_    =. ;
if  cd3_gd_ < 0 then    cd3_gd_ =. ;
if  NK__lymphs  < 0 then    NK__lymphs  =. ;
if  B_CELLS < 0 then    B_CELLS =. ;
if  cd4_cd25_   < 0 then    cd4_cd25_   =. ;
if  cd6_cd127_cd4_cd25__as___lymphs < 0 then    cd6_cd127_cd4_cd25__as___lymphs =. ;
if  cd4_cd25_cd6dim < 0 then    cd4_cd25_cd6dim =. ;
if  CD14__CD16_ < 0 then    CD14__CD16_ =. ;
if  CD14_CD16_  < 0 then    CD14_CD16_  =. ;
if  xx  < 0 then    xx  =. ;
if  mesa_th1_final_CD4_ < 0 then    mesa_th1_final_CD4_ =. ;
if  mesa_th1_final_CD8_ < 0 then    mesa_th1_final_CD8_ =. ;
if  th1_as__cd4 < 0 then    th1_as__cd4 =. ;
if  th2_as__cd4 < 0 then    th2_as__cd4 =. ;
if  th17_as__cd4    < 0 then    th17_as__cd4    =. ;
if  cd8IFNas__cd8   < 0 then    cd8IFNas__cd8   =. ;
if  cd8il4_as__cd8  < 0 then    cd8il4_as__cd8  =. ;
if  cd8il17_as__cd8 < 0 then    cd8il17_as__cd8 =. ;

label   cd4_mnas_mesa_final_CD4_    = ' CD4+ cells (as % lymphs)'   ;
```

```
label   CD4_CD45RA_ = ' cd4+ Naïve Cells (as % CD4+ cells)' ;
label   CD4_CD45RO_ = ' CD4+ Memory Cells (as % CD4+ Cells)'    ;
label   CD4_CD28_CD57_  = ' Immunosenescent Subpopulation of CD4+ Cells (as % of CD4+ Cells)'   ;
label   CD4_CD38_   = ' Activated CD4 Cells (as % CD4+ Cells)'  ;
label   CD4_CD28_   = ' Immunosenescent CD4+ Cells (as % of CD4+ Cells)'    ;
label   CD4_CD45RA_CD28_CD57__AS_CD4    = ' CD4+cd45RA re-expressing senescent cells (as %CD4+ cells)'
label   CD4_CD57_   = ' Measure of chronic immune activation on CD4+ cells' ;
label   cd8_mnas_mesa_final_CD8_    = ' cd8+ cells (as % lymphs)'   ;
label   CD8_CD45RA_ = ' cd8+ Naïve Cells (as % CD8+ cells)' ;
label   CD8_CD45RO_ = ' CD8+ Memory Cells (as % CD8+ Cells)'    ;
label   CD8_CD28_CD57_  = ' Immunosenescent Subpopulation CD8+ Cells (as % of CD8+ Cells)'  ;
label   CD8_CD38_   = ' Activated CD8 Cells (as % CD8+ Cells)'  ;
label   CD8_CD28_   = ' Immunosenescent CD8+ Cells (as % of CD8+ Cells)'    ;
label   CD8_CD45RA_CD28_CD57__AS_CD8    = ' cd8+cd45RA re-expressing senescent cells (as %CD8+ cells)'
label   CD8_CD57_   = ' Measure of chronic immune activation on CD8+ cells' ;
label   cd3_    = ' CD3+ Cells (as % Lymphs)'   ;
label   cd3_gd_ = ' gamma delta T cells (as % Lymphs)'  ;
label   NK__lymphs  = ' Natural Killer Cells (as % lymphs)' ;
label   B_CELLS = ' CD19+ Cells (As % lymphs)'  ;
label   cd4_cd25_   = ' T regulatory cells (as a % of lymphs)'  ;
label   cd6_cd127_cd4_cd25__as___lymphs = ' FoxP3-type T regulatory Cell (as % lymphs)' ;
label   cd4_cd25_cd6dim = ' Another reported measure of FoxP3-type T regulatory cells'  ;
label   CD14__CD16_ = ' Classic Monocytes'  ;
label   CD14_CD16_  = ' Intermediate Monocytes' ;
label   xx  = ' non-classic monocytes'  ;
label   mesa_th1_final_CD4_ = ' CD4+ cells (as % lymphs)'   ;
label   mesa_th1_final_CD8_ = ' cd8+ cells (as % lymphs)'   ;
label   th1_as__cd4 = ' cd4+ifn+ as % CD4+ (TH1)'   ;
label   th2_as__cd4 = ' cd4+il4+ as %CD4+ (Th2)'    ;
label   th17_as__cd4    = ' CD4+IL17+ as %CD4+ (IL17)'  ;
label   cd8IFNas__cd8   = ' cd8+ifn+ as %CD8+'  ;
label   cd8il4_as__cd8  = ' cd8+il4+ as %cd8+'  ;
label   cd8il17_as__cd8 = ' cd8+il17+ as %CD8+' ;

q=sum(xx,CD14_CD16_,CD14__CD16_);
CD14__CD16_2 = (CD14__CD16_/q)*100;
CD14_CD16_2 = (CD14_CD16_/q)*100;
xx2 = (xx/q)*100;


run;

proc sort data=check_1;
by idno;
run;

data lib.Lymphocytes_MESA_July_5_2018;
merge check_1 (in=ina) quat (in=inb);
by idno;

if ina;
if inb;

run;
```

```
proc means;
run;

proc sort data=check_1;
by idno;
run;
proc sort data=lib.cmv;
by idno;
run;

proc means;
where xx ne . and NK__lymphs ne .;
run;

data check;
merge check_1 lib.cmv;
by idno;
if cmv=-999 then cmv=.;
if cmv=-555 then cmv=960;
if cmv=-333 then cmv=0.01;
if cmv=-888then cmv=.;
log_cmv=log(cmv+1);

run;


proc means maxdec=2;
var
th1_as__cd4
th17_as__cd4
CD4_CD38_
CD8_CD38_
NK__lymphs
cd3_gd_
CD14__CD16_

th2_as__cd4
XX

cd4_cd25_
CD14_CD16_

CD4_CD45RA_
CD8_CD45RA_
CD4_CD28_
CD8_CD28_
CD4_CD45RO_
CD8_CD45RO_

cd4_mnas_mesa_final_CD4_
CD4_CD28_CD57_
CD4_CD45RA_CD28_CD57__AS_CD4
CD4_CD57_
cd8_mnas_mesa_final_CD8_
CD8_CD28_CD57_
CD8_CD45RA_CD28_CD57__AS_CD8
```

```
CD8_CD57_
cd3_
B_CELLS
cd6_cd127_cd4_cd25__as___lymphs
cd4_cd25_cd6dim
mesa_th1_final_CD4_
mesa_th1_final_CD8_
cd8IFNas__cd8
cd8il4_as__cd8
cd8il17_as__cd8
cmv
;
run;


proc sort data=check nodupkey;
by idno;
run;




/* Second, Assemble longitudinal data
   Divide lymphocyte subsets (in %) by standard deviations for scaling */

/*Covariates: smoking, diabetes, physical activity, and baseline body mass index.
These are the primary risk factors for hypertension.*/


data one_p;
merge check (in=ina) lib.Mesae1finallabel05072015 (keep=idno age1c gender1 race1c sbp1c ace1c aced1c c[illegible]
cursmk1 dm031c curalc1 bmi1c chol1 exercm1c educ1 il61 chol1 hdl1 sttn1c cmv1);
by idno;
if ina;
sbp=sbp1c;
dbp=dbp1c;
time=0;

if dm031c> 1 then diabetes=1;
else diabetes=0;

ace=max(ace1c,aced1c);
ccb=ccb1c;
diur=max(aced1c, diur1c, betad1c, vasod1c);
beta=max(betad1c, beta1c);
vaso=max(vaso1c, vasod1c);
if RACE1C=1 then r1=1;
else r1=0;
if RACE1C=2 then r2=1;
else r2=0;
if RACE1C=3 then r3=1;
else r3=0;
if RACE1C=4 then r4=1;
else r4=0;
any_ah=max(ace,ccb,diur,beta,vaso);
age=age1c;
if gender1=1 then male=1;
```

```
else male=0;

if any_ah=1 then sbp_adj=sbp+10;
else sbp_adj=sbp;
if educ1>6 then college=1;
else college=0;

cd4_mnas_mesa_final_CD4_    =   cd4_mnas_mesa_final_CD4_    /   11.0148293  ;
CD4_CD45RA_ =   CD4_CD45RA_ /   12.0481371  ;
CD4_CD45RO_ =   CD4_CD45RO_ /   13.4133922  ;
CD4_CD28_CD57_  =   CD4_CD28_CD57_  /   8.4565482   ;
LIVE_LYMPH_COUNT    =   LIVE_LYMPH_COUNT    /   7044.02 ;
CD4_CD38_   =   CD4_CD38_   /   12.1331635  ;
CD4_CD28_   =   CD4_CD28_   /   10.0446624  ;
CD4_CD45RA_CD28_CD57__AS_CD4    =   CD4_CD45RA_CD28_CD57__AS_CD4    /   5.3288475   ;
CD4_CD57_   =   CD4_CD57_   /   12.9488516  ;
cd8_mnas_mesa_final_CD8_    =   cd8_mnas_mesa_final_CD8_    /   9.3385893   ;
CD8_CD45RA_ =   CD8_CD45RA_ /   14.6668267  ;
CD8_CD45RO_ =   CD8_CD45RO_ /   10.6215264  ;
CD8_CD28_CD57_  =   CD8_CD28_CD57_  /   15.8827921  ;
LIVE_LYMPH_COUNT_8  =   LIVE_LYMPH_COUNT_8  /   7381.23 ;
CD8_CD38_   =   CD8_CD38_   /   12.1970917  ;
CD8_CD28_   =   CD8_CD28_   /   15.9305801  ;
CD8_CD45RA_CD28_CD57__AS_CD8    =   CD8_CD45RA_CD28_CD57__AS_CD8    /   14.2740251  ;
CD8_CD57_   =   CD8_CD57_   /   15.4380623  ;
cd3_    =   cd3_    /   13.6970536  ;
cd3_gd_ =   cd3_gd_ /   4.0324039   ;
NK__lymphs  =   NK__lymphs  /   5.6925374   ;
B_CELLS =   B_CELLS /   7.3817656   ;
cd4_cd25_   =   cd4_cd25_   /   7.1005486   ;
cd6_cd127_cd4_cd25__as___lymphs =   cd6_cd127_cd4_cd25__as___lymphs /   0.5234053   ;
cd4_cd25_cd6dim =   cd4_cd25_cd6dim /   3.7634147   ;
CD14__CD16_ =   CD14__CD16_ /   12.8157347  ;
CD14_CD16_  =   CD14_CD16_  /   4.9169505   ;
XX  =   XX  /   3.2611816   ;
mesa_th1_final_CD4_ =   mesa_th1_final_CD4_ /   13.5738544  ;
mesa_th1_final_CD8_ =   mesa_th1_final_CD8_ /   8.3438174   ;
th1_as__cd4 =   th1_as__cd4 /   8.9953262   ;
th2_as__cd4 =   th2_as__cd4 /   1.7477505   ;
th17_as__cd4    =   th17_as__cd4    /   1.4328386   ;
cd8IFNas__cd8   =   cd8IFNas__cd8   /   17.8840879  ;
cd8il4_as__cd8  =   cd8il4_as__cd8  /   4.9215664   ;
cd8il17_as__cd8 =   cd8il17_as__cd8 /   5.7672104   ;


CD14__CD16_3    =   CD14__CD16_2    /10.2084227     ;
CD14_CD16_3 =   CD14_CD16_2 /   7.1003813   ;
XX3 =   XX2 /   7.5327375 ;





run;

proc means;
var male;
```

```
run;

proc means data=one_p;
var age male r2 r3 r4 LIVE_LYMPH_COUNT sbp ace ccb diur beta vaso any;
run;

proc contents data=lib.mesadiet2;
run;
proc sort data=lib.mesadiet2;
by idno;
run;


data one;
merge one_p (in=ina) lib.mesadiet2 (keep=idno wine liquor beer);
by idno;
if ina;

if beer='OM' or wine ='OM' or liquor ='OM' then add_alc=1;
else if beer ne . then add_alc=0;

if curalc1 ne . then curalc1r=curalc1;
else if add_alc=1 then curalc1r=1;
else if add_alc=0 then curalc1r=0;
run;


proc means ;
var curalc1 curalc1r;
run;


proc freq;
tables wine liquor beer;
run;


data two;
merge check (in=ina) lib.Mesae2finallabel02132015 (keep=idno e12dyc age2c age1c  gender1 race1c sbp2c 
dm032c curalc2 bmi2c exercm2c);
by idno;
if ina;
sbp=sbp2c;
dbp=dbp2c;
time=e12dyc/365;
age=age2c;

ace=max(ace2c,aced2c);
ccb=ccb2c;
diur=max(aced2c, diur2c, betad2c, vasod2c);
beta=max(betad2c, beta2c);
vaso=max(vaso2c, vasod2c);
any_ah=max(ace,ccb,diur,beta,vaso);
if any_ah=1 then sbp_adj=sbp+10;
else sbp_adj=sbp;cd4_mnas_mesa_final_CD4_   =   cd4_mnas_mesa_final_CD4_    /   11.0148293  ;
CD4_CD45RA_ =   CD4_CD45RA_ /   12.0481371  ;
```

```
CD4_CD45RO_ =   CD4_CD45RO_ /   13.4133922  ;
CD4_CD28_CD57_  =   CD4_CD28_CD57_  /   8.4565482   ;
LIVE_LYMPH_COUNT    =   LIVE_LYMPH_COUNT    /   7044.02 ;
CD4_CD38_   =   CD4_CD38_   /   12.1331635  ;
CD4_CD28_   =   CD4_CD28_   /   10.0446624  ;
CD4_CD45RA_CD28_CD57__AS_CD4    =   CD4_CD45RA_CD28_CD57__AS_CD4    /   5.3288475   ;
CD4_CD57_   =   CD4_CD57_   /   12.9488516  ;
cd8_mnas_mesa_final_CD8_    =   cd8_mnas_mesa_final_CD8_    /   9.3385893   ;
CD8_CD45RA_ =   CD8_CD45RA_ /   14.6668267  ;
CD8_CD45RO_ =   CD8_CD45RO_ /   10.6215264  ;
CD8_CD28_CD57_  =   CD8_CD28_CD57_  /   15.8827921  ;
LIVE_LYMPH_COUNT_8  =   LIVE_LYMPH_COUNT_8  /   7381.23 ;
CD8_CD38_   =   CD8_CD38_   /   12.1970917  ;
CD8_CD28_   =   CD8_CD28_   /   15.9305801  ;
CD8_CD45RA_CD28_CD57__AS_CD8    =   CD8_CD45RA_CD28_CD57__AS_CD8    /   14.2740251  ;
CD8_CD57_   =   CD8_CD57_   /   15.4380623  ;
cd3_    =   cd3_    /   13.6970536  ;
cd3_gd_ =   cd3_gd_ /   4.0324039   ;
NK__lymphs  =   NK__lymphs  /   5.6925374   ;
B_CELLS =   B_CELLS /   7.3817656   ;
cd4_cd25_   =   cd4_cd25_   /   7.1005486   ;
cd6_cd127_cd4_cd25__as___lymphs =   cd6_cd127_cd4_cd25__as___lymphs /   0.5234053   ;
cd4_cd25_cd6dim =   cd4_cd25_cd6dim /   3.7634147   ;
CD14__CD16_ =   CD14__CD16_ /   12.8157347  ;
CD14_CD16_  =   CD14_CD16_  /   4.9169505   ;
XX  =   XX  /   3.2611816   ;
mesa_th1_final_CD4_ =   mesa_th1_final_CD4_ /   13.5738544  ;
mesa_th1_final_CD8_ =   mesa_th1_final_CD8_ /   8.3438174   ;
th1_as__cd4 =   th1_as__cd4 /   8.9953262   ;
th2_as__cd4 =   th2_as__cd4 /   1.7477505   ;
th17_as__cd4    =   th17_as__cd4    /   1.4328386   ;
cd8IFNas__cd8   =   cd8IFNas__cd8   /   17.8840879  ;
cd8il4_as__cd8  =   cd8il4_as__cd8  /   4.9215664   ;
cd8il17_as__cd8 =   cd8il17_as__cd8 /   5.7672104   ;


CD14__CD16_3    =   CD14__CD16_2    /10.2084227     ;
CD14_CD16_3 =   CD14_CD16_2 /   7.1003813   ;
XX3 =   XX2 /   7.5327375 ;

run;

data three;
merge check (in=ina) lib.Mesae3finallabel02132015 (keep=idno  e13dyc age3c age1c gender1 race1c sbp3c [illegible]
cursmk3 dm033c curalc3 bmi3c dbp3c exercm3c);
by idno;
if ina;
sbp=sbp3c;
dbp=dbp3c;
time=e13dyc/365;
age=age3c;


ace=max(ace3c,aced3c);
ccb=ccb3c;
diur=max(aced3c, diur3c, betad3c, vasod3c);
```

```
beta=max(betad3c, beta3c);
vaso=max(vaso3c, vasod3c);
any_ah=max(ace,ccb,diur,beta,vaso);
if any_ah=1 then sbp_adj=sbp+10;
else sbp_adj=sbp;cd4_mnas_mesa_final_CD4_   =   cd4_mnas_mesa_final_CD4_    /   11.0148293  ;
CD4_CD45RA_ =   CD4_CD45RA_ /   12.0481371  ;
CD4_CD45RO_ =   CD4_CD45RO_ /   13.4133922  ;
CD4_CD28_CD57_  =   CD4_CD28_CD57_  /   8.4565482   ;
LIVE_LYMPH_COUNT    =   LIVE_LYMPH_COUNT    /   7044.02 ;
CD4_CD38_   =   CD4_CD38_   /   12.1331635  ;
CD4_CD28_   =   CD4_CD28_   /   10.0446624  ;
CD4_CD45RA_CD28_CD57__AS_CD4    =   CD4_CD45RA_CD28_CD57__AS_CD4    /   5.3288475   ;
CD4_CD57_   =   CD4_CD57_   /   12.9488516  ;
cd8_mnas_mesa_final_CD8_    =   cd8_mnas_mesa_final_CD8_    /   9.3385893   ;
CD8_CD45RA_ =   CD8_CD45RA_ /   14.6668267  ;
CD8_CD45RO_ =   CD8_CD45RO_ /   10.6215264  ;
CD8_CD28_CD57_  =   CD8_CD28_CD57_  /   15.8827921  ;
LIVE_LYMPH_COUNT_8  =   LIVE_LYMPH_COUNT_8  /   7381.23 ;
CD8_CD38_   =   CD8_CD38_   /   12.1970917  ;
CD8_CD28_   =   CD8_CD28_   /   15.9305801  ;
CD8_CD45RA_CD28_CD57__AS_CD8    =   CD8_CD45RA_CD28_CD57__AS_CD8    /   14.2740251  ;
CD8_CD57_   =   CD8_CD57_   /   15.4380623  ;
cd3_    =   cd3_    /   13.6970536  ;
cd3_gd_ =   cd3_gd_ /   4.0324039   ;
NK__lymphs  =   NK__lymphs  /   5.6925374   ;
B_CELLS =   B_CELLS /   7.3817656   ;
cd4_cd25_   =   cd4_cd25_   /   7.1005486   ;
cd6_cd127_cd4_cd25__as___lymphs =   cd6_cd127_cd4_cd25__as___lymphs /   0.5234053   ;
cd4_cd25_cd6dim =   cd4_cd25_cd6dim /   3.7634147   ;
CD14__CD16_ =   CD14__CD16_ /   12.8157347  ;
CD14_CD16_  =   CD14_CD16_  /   4.9169505   ;
XX  =   XX  /   3.2611816   ;
mesa_th1_final_CD4_ =   mesa_th1_final_CD4_ /   13.5738544  ;
mesa_th1_final_CD8_ =   mesa_th1_final_CD8_ /   8.3438174   ;
th1_as__cd4 =   th1_as__cd4 /   8.9953262   ;
th2_as__cd4 =   th2_as__cd4 /   1.7477505   ;
th17_as__cd4    =   th17_as__cd4    /   1.4328386   ;
cd8IFNas__cd8   =   cd8IFNas__cd8   /   17.8840879  ;
cd8il4_as__cd8  =   cd8il4_as__cd8  /   4.9215664   ;
cd8il17_as__cd8 =   cd8il17_as__cd8 /   5.7672104   ;

CD14__CD16_3    =   CD14__CD16_2    /10.2084227     ;
CD14_CD16_3 =   CD14_CD16_2 /   7.1003813   ;
XX3 =   XX2 /   7.5327375 ;

run;

data four;
merge check (in=ina) lib.Mesae4finallabel02172015 (keep=idno e14dyc age4c gender1 race1c sbp4c ace4c a
cursmk4 dm034c curalc4 bmi4c dbp4c);
by idno;
if ina;
sbp=sbp4c;
dbp=dbp4c;
time=e14dyc/365;
age=age4c;
```

```
cd4_mnas_mesa_final_CD4_    =   cd4_mnas_mesa_final_CD4_    /   11.0148293  ;
CD4_CD45RA_ =   CD4_CD45RA_ /   12.0481371  ;
CD4_CD45RO_ =   CD4_CD45RO_ /   13.4133922  ;
CD4_CD28_CD57_  =   CD4_CD28_CD57_  /   8.4565482   ;
LIVE_LYMPH_COUNT    =   LIVE_LYMPH_COUNT    /   7044.02 ;
CD4_CD38_   =   CD4_CD38_   /   12.1331635  ;
CD4_CD28_   =   CD4_CD28_   /   10.0446624  ;
CD4_CD45RA_CD28_CD57__AS_CD4    =   CD4_CD45RA_CD28_CD57__AS_CD4    /   5.3288475   ;
CD4_CD57_   =   CD4_CD57_   /   12.9488516  ;
cd8_mnas_mesa_final_CD8_    =   cd8_mnas_mesa_final_CD8_    /   9.3385893   ;
CD8_CD45RA_ =   CD8_CD45RA_ /   14.6668267  ;
CD8_CD45RO_ =   CD8_CD45RO_ /   10.6215264  ;
CD8_CD28_CD57_  =   CD8_CD28_CD57_  /   15.8827921  ;
LIVE_LYMPH_COUNT_8  =   LIVE_LYMPH_COUNT_8  /   7381.23 ;
CD8_CD38_   =   CD8_CD38_   /   12.1970917  ;
CD8_CD28_   =   CD8_CD28_   /   15.9305801  ;
CD8_CD45RA_CD28_CD57__AS_CD8    =   CD8_CD45RA_CD28_CD57__AS_CD8    /   14.2740251  ;
CD8_CD57_   =   CD8_CD57_   /   15.4380623  ;
cd3_    =   cd3_    /   13.6970536  ;
cd3_gd_ =   cd3_gd_ /   4.0324039   ;
NK__lymphs  =   NK__lymphs  /   5.6925374   ;
B_CELLS =   B_CELLS /   7.3817656   ;
cd4_cd25_   =   cd4_cd25_   /   7.1005486   ;
cd6_cd127_cd4_cd25__as___lymphs =   cd6_cd127_cd4_cd25__as___lymphs /   0.5234053   ;
cd4_cd25_cd6dim =   cd4_cd25_cd6dim /   3.7634147   ;
CD14__CD16_ =   CD14__CD16_ /   12.8157347  ;
CD14_CD16_  =   CD14_CD16_  /   4.9169505   ;
XX  =   XX  /   3.2611816   ;
mesa_th1_final_CD4_ =   mesa_th1_final_CD4_ /   13.5738544  ;
mesa_th1_final_CD8_ =   mesa_th1_final_CD8_ /   8.3438174   ;
th1_as__cd4 =   th1_as__cd4 /   8.9953262   ;
th2_as__cd4 =   th2_as__cd4 /   1.7477505   ;
th17_as__cd4    =   th17_as__cd4    /   1.4328386   ;
cd8IFNas__cd8   =   cd8IFNas__cd8   /   17.8840879  ;
cd8il4_as__cd8  =   cd8il4_as__cd8  /   4.9215664   ;
cd8il17_as__cd8 =   cd8il17_as__cd8 /   5.7672104   ;


CD14__CD16_3    =   CD14__CD16_2    /10.2084227     ;
CD14_CD16_3 =   CD14_CD16_2 /   7.1003813   ;
XX3 =   XX2 /   7.5327375 ;


ace=max(ace4c,aced4c);
ccb=ccb4c;
diur=max(aced4c, diur4c, betad4c, vasod4c);
beta=max(betad4c, beta4c);
vaso=max(vaso4c, vasod4c);
any_ah=max(ace,ccb,diur,beta,vaso);
if any_ah=1 then sbp_adj=sbp+10;
else sbp_adj=sbp;
run;

data five;
merge check (in=ina) lib.Mesae5_finallabel_20141107 (keep=idno e15dyc age5c age1c gender1 race1c sbp5c
cursmk5 dm035c curalc5 bmi5c dbp5c exercm5c);
```

```
by idno;
if ina;
sbp=sbp5c;
dbp=dbp5c;
time=e15dyc/365;
age=age5c;
group=1;
ace=max(ace5c,aced5c);
ccb=ccb5c;
diur=max(aced5c, diur5c, betad5c, vasod5c);
beta=max(betad5c, beta5c);
vaso=max(vaso5c, vasod5c);
any_ah=max(ace,ccb,diur,beta,vaso);
if any_ah=1 then sbp_adj=sbp+10;
else sbp_adj=sbp;cd4_mnas_mesa_final_CD4_   =   cd4_mnas_mesa_final_CD4_    /   11.0148293  ;
CD4_CD45RA_ =   CD4_CD45RA_ /   12.0481371  ;
CD4_CD45RO_ =   CD4_CD45RO_ /   13.4133922  ;
CD4_CD28_CD57_  =   CD4_CD28_CD57_  /   8.4565482   ;
LIVE_LYMPH_COUNT    =   LIVE_LYMPH_COUNT    /   7044.02 ;
CD4_CD38_   =   CD4_CD38_   /   12.1331635  ;
CD4_CD28_   =   CD4_CD28_   /   10.0446624  ;
CD4_CD45RA_CD28_CD57__AS_CD4    =   CD4_CD45RA_CD28_CD57__AS_CD4    /   5.3288475   ;
CD4_CD57_   =   CD4_CD57_   /   12.9488516  ;
cd8_mnas_mesa_final_CD8_    =   cd8_mnas_mesa_final_CD8_    /   9.3385893   ;
CD8_CD45RA_ =   CD8_CD45RA_ /   14.6668267  ;
CD8_CD45RO_ =   CD8_CD45RO_ /   10.6215264  ;
CD8_CD28_CD57_  =   CD8_CD28_CD57_  /   15.8827921  ;
LIVE_LYMPH_COUNT_8  =   LIVE_LYMPH_COUNT_8  /   7381.23 ;
CD8_CD38_   =   CD8_CD38_   /   12.1970917  ;
CD8_CD28_   =   CD8_CD28_   /   15.9305801  ;
CD8_CD45RA_CD28_CD57__AS_CD8    =   CD8_CD45RA_CD28_CD57__AS_CD8    /   14.2740251  ;
CD8_CD57_   =   CD8_CD57_   /   15.4380623  ;
cd3_    =   cd3_    /   13.6970536  ;
cd3_gd_ =   cd3_gd_ /   4.0324039   ;
NK__lymphs  =   NK__lymphs  /   5.6925374   ;
B_CELLS =   B_CELLS /   7.3817656   ;
cd4_cd25_   =   cd4_cd25_   /   7.1005486   ;
cd6_cd127_cd4_cd25__as___lymphs =   cd6_cd127_cd4_cd25__as___lymphs /   0.5234053   ;
cd4_cd25_cd6dim =   cd4_cd25_cd6dim /   3.7634147   ;
CD14__CD16_ =   CD14__CD16_ /   12.8157347  ;
CD14_CD16_  =   CD14_CD16_  /   4.9169505   ;
XX  =   XX  /   3.2611816   ;
mesa_th1_final_CD4_ =   mesa_th1_final_CD4_ /   13.5738544  ;
mesa_th1_final_CD8_ =   mesa_th1_final_CD8_ /   8.3438174   ;
th1_as__cd4 =   th1_as__cd4 /   8.9953262   ;
th2_as__cd4 =   th2_as__cd4 /   1.7477505   ;
th17_as__cd4    =   th17_as__cd4    /   1.4328386   ;
cd8IFNas__cd8   =   cd8IFNas__cd8   /   17.8840879  ;
cd8il4_as__cd8  =   cd8il4_as__cd8  /   4.9215664   ;
cd8il17_as__cd8 =   cd8il17_as__cd8 /   5.7672104   ;

CD14__CD16_3    =   CD14__CD16_2    /10.2084227     ;
CD14_CD16_3 =   CD14_CD16_2 /   7.1003813   ;
XX3 =   XX2 /   7.5327375 ;
run;
```

```
data a;
set one two;
run;

proc means;
run;

data b;
set a three;
run;

data c;
set b four;
run;

data d (drop=exercm1c educ1 college);;
set c five;
if sbp ne .;

if RACE1C=1 then r1=1;
else r1=0;
if RACE1C=2 then r2=1;
else r2=0;
if RACE1C=3 then r3=1;
else r3=0;
if RACE1C=4 then r4=1;
else r4=0;

if gender1=1 then male=1;
else male=0;


age2=age1c/9.8305287;
time2=time/3.1773682;

monocyte1= CD14__CD16_ /12.36 ;
monocyte2=CD14_CD16_/4.32 ;
monocyte3=xx/3.29 ;

age_sq=age*age;

chinese = r2;
black=r3;
hispanic = r4;

cursmk=max(cursmk1, cursmk2, cursmk3, cursmk4, cursmk5);
dm03=max(dm031c, dm032c, dm033c, dm034c, dm035c);
curalc=max(curalc1r, curalc2, curalc3, curalc4, curalc5);
 curalc_new=max(add_alc, curalc2, curalc3, curalc4, curalc5);
curalc_old=max(curalc1, curalc2, curalc3, curalc4, curalc5);
bmi=max(bmi1c, bmi2c, bmi3c, bmi4c, bmi5c);
exercm=max(exercm1c,exercm2c,exercm3c,exercm4c,exercm5c);

if dm03 >1 then diabetes=1;
else diabetes = 0;
```

```
mesa_th1_final_CD8_SD=mesa_th1_final_CD8_/8.849411;
mesa_th1_final_CD4_SD= mesa_th1_final_CD4_/12.6115761;
if cursmk=. then cursmk=0;
run;
proc means;
var cursmk cursmk1 cursmk2 cursmk3 cursmk4 cursmk5 exercm diabetes;
run;

proc freq;
tables dm03 ;

run;

proc freq;
tables race1c;
where time=0;
run;

data pp (keep=idno exercm1c educ1 college add_alc);
merge check (in=ina) one;
by idno;
if ina;

if educ1>6 then college=1;
else college=0;

run;

proc means;
run;

proc sort data=d;
by idno;
run;

data d3;
merge pp d (drop=add_alc);
by idno;

run;

proc means;
run;


proc sort data=d3;
by idno;
run;

data e;
merge d3 (in=ina) quat(in=inb);
by idno;
if ina;
if inb;
```

```
ratio=cd4_cd25_/th17_as__cd4;
run;

proc means data=e;
var cursmk cursmk1 cursmk2 cursmk3 cursmk4 cursmk5 samp_weight;
run;

proc means;
var age   male chinese black hispanic cursmk sbp dbp time
diabetes
curalc
bmi cd4_mnas_mesa_final_CD4_
CD4_CD45RA_
CD4_CD45RO_
CD4_CD28_CD57_
CD4_CD38_
CD4_CD28_
CD4_CD45RA_CD28_CD57__AS_CD4
CD4_CD57_
cd8_mnas_mesa_final_CD8_
CD8_CD45RA_
CD8_CD45RO_
CD8_CD28_CD57_
CD8_CD38_
CD8_CD28_
CD8_CD45RA_CD28_CD57__AS_CD8
CD8_CD57_
cd3_
cd3_gd_
NK__lymphs
B_CELLS
cd4_cd25_
cd6_cd127_cd4_cd25__as___lymphs
cd4_cd25_cd6dim
CD14__CD16_
CD14_CD16_
xx
mesa_th1_final_CD4_
mesa_th1_final_CD8_
th1_as__cd4
th2_as__cd4
th17_as__cd4
cd8IFNas__cd8
cd8il4_as__cd8
cd8il17_as__cd8 exercm1c exercm
;
run;




data d2 (keep =idno study age   male chinese black hispanic cursmk sbp dbp time
diabetes
curalc
bmi cd4_mnas_mesa_final_CD4_
```

```
CD4_CD45RA_
CD4_CD45RO_
CD4_CD28_CD57_
CD4_CD38_
CD4_CD28_
CD4_CD45RA_CD28_CD57__AS_CD4
CD4_CD57_
cd8_mnas_mesa_final_CD8_
CD8_CD45RA_
CD8_CD45RO_
CD8_CD28_CD57_
CD8_CD38_
CD8_CD28_
CD8_CD45RA_CD28_CD57__AS_CD8
CD8_CD57_
cd3_
cd3_gd_
NK__lymphs
B_CELLS
cd4_cd25_
cd6_cd127_cd4_cd25__as___lymphs
cd4_cd25_cd6dim
CD14__CD16_
CD14_CD16_
xx
mesa_th1_final_CD4_
mesa_th1_final_CD8_
th1_as__cd4
th2_as__cd4
th17_as__cd4
cd8IFNas__cd8
cd8il4_as__cd8
cd8il17_as__cd8 );
set e;

run;




/* Third, now that the data is assembled, automate a series of mixed models to estimate mean
   Systolic blood pressure across 10 years of follow-up */






data table;
run;
proc mixed data=e METHOD=REML EMPIRICAL;
class idno;
model sbp_adj = ratio age   male r2 r3 r4  cursmk
exercm1c college diabetes log_cmv

bmi diabetes log_cmv
```

```
 / cl ;
random Intercept/subject=idno;
repeated /subject=idno; weight samp_weight;

ods output solutionF=sol;
run;


data sol2;
set sol;

if _N_ = 2;
run;

data table;
set sol2;
run;

proc mixed data=e METHOD=REML EMPIRICAL;
class idno;
model sbp_adj = cd4_mnas_mesa_final_CD4_ age   male r2 r3 r4  cursmk
exercm1c college diabetes log_cmv

bmi diabetes log_cmv

 / cl ;
random Intercept/subject=idno;
repeated /subject=idno; weight samp_weight;

ods output solutionF=sol;
run;


data sol2;
set sol;

if _N_ = 2;
run;

data table;
set sol2;
run;


proc mixed data=e METHOD=REML EMPIRICAL;
class idno;
model sbp_adj = CD4_CD45RA_
 age   male r2 r3 r4  cursmk
exercm1c college diabetes log_cmv diabetes log_cmv

bmi
 / cl ;
random Intercept/subject=idno;
repeated /subject=idno; weight samp_weight;

ods output solutionF=sol;
```

```
run;


data sol2;
set sol;

if _N_ = 2;
run;

data table;
set table sol2;
run;

proc mixed data=e METHOD=REML EMPIRICAL;
class idno;
model sbp_adj =CD4_CD45RO_
 age   male r2 r3 r4  cursmk
exercm1c college diabetes log_cmv

bmi
 / cl ;
random Intercept/subject=idno;
repeated /subject=idno; weight samp_weight;

ods output solutionF=sol;
run;


data sol2;
set sol;

if _N_ = 2;
run;

data table;
set table sol2;
run;

proc mixed data=e METHOD=REML EMPIRICAL;
class idno;
model sbp_adj = CD4_CD28_CD57_
 age   male r2 r3 r4  cursmk
exercm1c college diabetes log_cmv

bmi
 / cl ;
random Intercept/subject=idno;
repeated /subject=idno; weight samp_weight;

ods output solutionF=sol;
run;


data sol2;
set sol;
```

```
if _N_ = 2;
run;

data table;
set table sol2;
run;

proc mixed data=e METHOD=REML EMPIRICAL;
class idno;
model sbp_adj =CD4_CD38_
 age   male r2 r3 r4  cursmk
exercm1c college diabetes log_cmv

bmi
 / cl ;
random Intercept/subject=idno;
repeated /subject=idno; weight samp_weight;

ods output solutionF=sol;
run;


data sol2;
set sol;

if _N_ = 2;
run;

data table;
set table sol2;
run;

proc mixed data=e METHOD=REML EMPIRICAL;
class idno;
model sbp_adj = CD4_CD28_
 age   male r2 r3 r4  cursmk
exercm1c college diabetes log_cmv

bmi
 / cl ;
random Intercept/subject=idno;
repeated /subject=idno; weight samp_weight;

ods output solutionF=sol;
run;


data sol2;
set sol;

if _N_ = 2;
run;

data table;
set table sol2;
run;
```

```
proc mixed data=e METHOD=REML EMPIRICAL;
class idno;
model sbp_adj = CD4_CD45RA_CD28_CD57__AS_CD4
  age   male r2 r3 r4  cursmk
exercm1c college diabetes log_cmv

bmi
 / cl ;
random Intercept/subject=idno;
repeated /subject=idno; weight samp_weight;

ods output solutionF=sol;
run;


data sol2;
set sol;

if _N_ = 2;
run;

data table;
set table sol2;
run;

proc mixed data=e METHOD=REML EMPIRICAL;
class idno;
model sbp_adj = CD4_CD57_
 age   male r2 r3 r4  cursmk
exercm1c college diabetes log_cmv

bmi
 / cl ;
random Intercept/subject=idno;
repeated /subject=idno; weight samp_weight;

ods output solutionF=sol;
run;


data sol2;
set sol;

if _N_ = 2;
run;

data table;
set table sol2;
run;

proc mixed data=e METHOD=REML EMPIRICAL;
class idno;
model sbp_adj =cd8_mnas_mesa_final_CD8_
age   male r2 r3 r4  cursmk
exercm1c college diabetes log_cmv
```

```
bmi
 / cl ;
random Intercept/subject=idno;
repeated /subject=idno; weight samp_weight;

ods output solutionF=sol;
run;


data sol2;
set sol;

if _N_ = 2;
run;

data table;
set table sol2;
run;

proc mixed data=e METHOD=REML EMPIRICAL;
class idno;
model sbp_adj =CD8_CD45RA_
 age   male r2 r3 r4  cursmk
exercm1c college diabetes log_cmv

bmi
 / cl ;
random Intercept/subject=idno;
repeated /subject=idno; weight samp_weight;

ods output solutionF=sol;
run;


data sol2;
set sol;

if _N_ = 2;
run;

data table;
set table sol2;
run;

proc mixed data=e METHOD=REML EMPIRICAL;
class idno;
model sbp_adj = CD8_CD45RO_
 age   male r2 r3 r4  cursmk
exercm1c college diabetes log_cmv

bmi
 / cl ;
random Intercept/subject=idno;
repeated /subject=idno; weight samp_weight;
```

```
ods output solutionF=sol;
run;


data sol2;
set sol;

if _N_ = 2;
run;

data table;
set table sol2;
run;

proc mixed data=e METHOD=REML EMPIRICAL;
class idno;
model sbp_adj = CD8_CD28_CD57_
  age   male r2 r3 r4  cursmk
exercm1c college diabetes log_cmv

bmi
 / cl ;
random Intercept/subject=idno;
repeated /subject=idno; weight samp_weight;

ods output solutionF=sol;
run;


data sol2;
set sol;

if _N_ = 2;
run;

data table;
set table sol2;
run;

proc mixed data=e METHOD=REML EMPIRICAL;
class idno;
model sbp_adj = CD8_CD38_
age   male r2 r3 r4  cursmk
exercm1c college diabetes log_cmv

bmi
 / cl ;
random Intercept/subject=idno;
repeated /subject=idno; weight samp_weight;

ods output solutionF=sol;
run;


data sol2;
set sol;
```

```
if _N_ = 2;
run;

data table;
set table sol2;
run;

proc mixed data=e METHOD=REML EMPIRICAL;
class idno;
model sbp_adj = CD8_CD28_
age   male r2 r3 r4  cursmk
exercm1c college diabetes log_cmv

bmi
 / cl ;
random Intercept/subject=idno;
repeated /subject=idno; weight samp_weight;

ods output solutionF=sol;
run;


data sol2;
set sol;

if _N_ = 2;
run;

data table;
set table sol2;
run;

proc mixed data=e METHOD=REML EMPIRICAL;
class idno;
model sbp_adj = CD8_CD45RA_CD28_CD57__AS_CD8
 age   male r2 r3 r4  cursmk
exercm1c college diabetes log_cmv

bmi
 / cl ;
random Intercept/subject=idno;
repeated /subject=idno; weight samp_weight;

ods output solutionF=sol;
run;


data sol2;
set sol;

if _N_ = 2;
run;

data table;
set table sol2;
```

```
run;

proc mixed data=e METHOD=REML EMPIRICAL;
class idno;
model sbp_adj = CD8_CD57_
age   male r2 r3 r4  cursmk
exercm1c college diabetes log_cmv

bmi
 / cl ;
random Intercept/subject=idno;
repeated /subject=idno; weight samp_weight;

ods output solutionF=sol;
run;


data sol2;
set sol;

if _N_ = 2;
run;

data table;
set table sol2;
run;

proc mixed data=e METHOD=REML EMPIRICAL;
class idno;
model sbp_adj =cd3_
age   male r2 r3 r4  cursmk
exercm1c college diabetes log_cmv

bmi
 / cl ;
random Intercept/subject=idno;
repeated /subject=idno; weight samp_weight;

ods output solutionF=sol;
run;


data sol2;
set sol;

if _N_ = 2;
run;

data table;
set table sol2;
run;

proc mixed data=e METHOD=REML EMPIRICAL;
class idno;
model sbp_adj = cd3_gd_
 age   male r2 r3 r4  cursmk
```

```
exercm1c college diabetes log_cmv

bmi
 / cl ;
random Intercept/subject=idno;
repeated /subject=idno; weight samp_weight;

ods output solutionF=sol;
run;


data sol2;
set sol;

if _N_ = 2;
run;

data table;
set table sol2;
run;

proc mixed data=e METHOD=REML EMPIRICAL;
class idno;
model sbp_adj = NK__lymphs
  age    male r2 r3 r4  cursmk
exercm1c college diabetes log_cmv

bmi
 / cl ;
random Intercept/subject=idno;
repeated /subject=idno; weight samp_weight;

ods output solutionF=sol;
run;


data sol2;
set sol;

if _N_ = 2;
run;

data table;
set table sol2;
run;




proc mixed data=e METHOD=REML EMPIRICAL;
class idno;
model sbp_adj =B_CELLS
 age   male r2 r3 r4  cursmk
exercm1c college diabetes log_cmv

bmi
```

```
 / cl ;
random Intercept/subject=idno;
repeated /subject=idno; weight samp_weight;

ods output solutionF=sol;
run;


data sol2;
set sol;

if _N_ = 2;
run;

data table;
set table sol2;
run;


proc mixed data=e METHOD=REML EMPIRICAL;
class idno;
model sbp_adj =cd4_cd25_


  age   male r2 r3 r4  cursmk
exercm1c college diabetes log_cmv

bmi
 / cl ;
random Intercept/subject=idno;
repeated /subject=idno; weight samp_weight; weight samp_weight;

ods output solutionF=sol;
run;


data sol2;
set sol;

if _N_ = 2;
run;

data table;
set table sol2;
run;

proc mixed data=e METHOD=REML EMPIRICAL;
class idno;
model sbp_adj = cd6_cd127_cd4_cd25__as___lymphs

  age   male r2 r3 r4  cursmk
exercm1c college diabetes log_cmv

bmi
 / cl ;
random Intercept/subject=idno;
```

```
repeated /subject=idno; weight samp_weight;

ods output solutionF=sol;
run;


data sol2;
set sol;

if _N_ = 2;
run;

data table;
set table sol2;
run;
proc mixed data=e METHOD=REML EMPIRICAL;
class idno;
model sbp_adj = cd4_cd25_cd6dim

  age   male r2 r3 r4  cursmk
exercm1c college diabetes log_cmv

bmi
 / cl ;
random Intercept/subject=idno;
repeated /subject=idno; weight samp_weight;

ods output solutionF=sol;
run;


data sol2;
set sol;

if _N_ = 2;
run;

data table;
set table sol2;
run;
proc mixed data=e METHOD=REML EMPIRICAL;
class idno;
model sbp_adj = CD14__CD16_

  age   male r2 r3 r4  cursmk
exercm1c college diabetes log_cmv

bmi
 / cl ;
random Intercept/subject=idno;
repeated /subject=idno; weight samp_weight;

ods output solutionF=sol;
run;
```

```
data sol2;
set sol;

if _N_ = 2;
run;

data table;
set table sol2;
run;
proc mixed data=e METHOD=REML EMPIRICAL;
class idno;
model sbp_adj = CD14_CD16_

  age   male r2 r3 r4  cursmk
exercm1c college diabetes log_cmv

bmi
 / cl ;
random Intercept/subject=idno;
repeated /subject=idno; weight samp_weight;

ods output solutionF=sol;
run;


data sol2;
set sol;

if _N_ = 2;
run;

data table;
set table sol2;
run;
proc mixed data=e METHOD=REML EMPIRICAL;
class idno;
model sbp_adj = xx

  age   male r2 r3 r4  cursmk
exercm1c college diabetes log_cmv

bmi
 / cl ;
random Intercept/subject=idno;
repeated /subject=idno; weight samp_weight;

ods output solutionF=sol;
run;


data sol2;
set sol;

if _N_ = 2;
run;
```

```
data table;
set table sol2;
run;
proc mixed data=e METHOD=REML EMPIRICAL;
class idno;
model sbp_adj = mesa_th1_final_CD4_

  age   male r2 r3 r4  cursmk
exercm1c college diabetes log_cmv

bmi
 / cl ;
random Intercept/subject=idno;
repeated /subject=idno; weight samp_weight;

ods output solutionF=sol;
run;


data sol2;
set sol;

if _N_ = 2;
run;

data table;
set table sol2;
run;
proc mixed data=e METHOD=REML EMPIRICAL;
class idno;
model sbp_adj = mesa_th1_final_CD8_

  age   male r2 r3 r4  cursmk
exercm1c college diabetes log_cmv

bmi
 / cl ;
random Intercept/subject=idno;
repeated /subject=idno; weight samp_weight;

ods output solutionF=sol;
run;


data sol2;
set sol;

if _N_ = 2;
run;

data table;
set table sol2;
run;


proc mixed data=e METHOD=REML EMPIRICAL;
```

```
class idno;
model sbp_adj = th1_as__cd4


  age   male r2 r3 r4  cursmk
exercm1c college diabetes log_cmv

bmi
 / cl ;
random Intercept/subject=idno;
repeated /subject=idno; weight samp_weight;
ods output solutionF=sol;
run;


data sol2;
set sol;

if _N_ = 2;
run;

data table;
set table sol2;
run;




proc mixed data=e METHOD=REML EMPIRICAL;
class idno;
model sbp_adj =th2_as__cd4

  age   male r2 r3 r4  cursmk
exercm1c college diabetes log_cmv

bmi
 / cl ;
random Intercept/subject=idno;
repeated /subject=idno; weight samp_weight;
ods output solutionF=sol;
run;


data sol2;
set sol;

if _N_ = 2;
run;

data table;
set table sol2;
run;
proc mixed data=e METHOD=REML EMPIRICAL;
class idno;
model sbp_adj = th17_as__cd4

  age   male r2 r3 r4  cursmk
```

```
exercm1c college diabetes log_cmv

bmi
 / cl ;
random Intercept/subject=idno;
repeated /subject=idno; weight samp_weight;
ods output solutionF=sol;
run;


data sol2;
set sol;

if _N_ = 2;
run;

data table;
set table sol2;
run;


proc mixed data=e METHOD=REML EMPIRICAL;
class idno;
model sbp_adj = cd8IFNas__cd8
  age   male r2 r3 r4  cursmk
exercm1c college diabetes log_cmv

bmi
 / cl ;
random Intercept/subject=idno;
repeated /subject=idno; weight samp_weight;

ods output solutionF=sol;
run;


data sol2;
set sol;

if _N_ = 2;
run;

data table;
set table sol2;
run;

proc mixed data=e METHOD=REML EMPIRICAL;
class idno;
model sbp_adj = cd8il4_as__cd8
  age   male r2 r3 r4  cursmk
exercm1c college diabetes log_cmv

bmi
 / cl ;
random Intercept/subject=idno;
repeated /subject=idno; weight samp_weight;
```

```
ods output solutionF=sol;
run;


data sol2;
set sol;

if _N_ = 2;
run;

data table;
set table sol2;
run;

proc mixed data=e METHOD=REML EMPIRICAL;
class idno;
model sbp_adj = cd8il17_as__cd8
  age   male r2 r3 r4  cursmk
exercm1c college diabetes log_cmv

bmi
 / cl ;
random Intercept/subject=idno;
repeated /subject=idno; weight samp_weight;

ods output solutionF=sol;
run;


data sol2;
set sol;

if _N_ = 2;
run;

data table;
set table sol2;
run;


proc mixed data=e METHOD=REML EMPIRICAL;
class idno;
model sbp_adj = CD14_CD16_2
  age   male r2 r3 r4  cursmk
exercm1c college diabetes log_cmv

 / cl ;

repeated /subject=idno;  weight samp_weight;

ods output solutionF=sol;
run;


data sol2;
```

```
set sol;

if _N_ = 2;
run;

data table;
set table sol2;
run;




proc mixed data=e METHOD=REML EMPIRICAL;
class idno;
model sbp_adj= xx2
  age   male r2 r3 r4  cursmk
exercm1c college diabetes log_cmv

bmi
 / cl ;

repeated /subject=idno;  weight samp_weight;

ods output solutionF=sol;
run;


data sol2;
set sol;

if _N_ = 2;
run;








proc mixed data=e METHOD=REML EMPIRICAL;
class idno;
model sbp_adj = CD14__CD16_2
  age   male r2 r3 r4  cursmk
exercm1c college diabetes log_cmv

 / cl ;
repeated /subject=idno;  weight samp_weight;

ods output solutionF=sol;
run;


data sol2;
set sol;

if _N_ = 2;
run;
```

```
data table;
set table sol2;
run;



proc mixed data=e METHOD=REML EMPIRICAL;
class idno;
model sbp_adj = CD14_CD16_3
  age   male r2 r3 r4  cursmk
exercm1c college diabetes log_cmv

 / cl ;

repeated /subject=idno;  weight samp_weight;

ods output solutionF=sol;
run;


data sol2;
set sol;

if _N_ = 2;
run;

data table;
set table sol2;
run;



proc mixed data=e METHOD=REML EMPIRICAL;
class idno;
model sbp_adj= xx3
  age   male r2 r3 r4  cursmk
exercm1c college diabetes log_cmv

bmi
 / cl ;

repeated /subject=idno;  weight samp_weight;

ods output solutionF=sol;
run;


data sol2;
set sol;

if _N_ = 2;
run;

data table;
set table sol2;
```

```
run;





proc mixed data=e METHOD=REML EMPIRICAL;
class idno;
model sbp_adj = CD14__CD16_3
  age   male r2 r3 r4  cursmk
exercm1c college diabetes log_cmv

 / cl ;
repeated /subject=idno;  weight samp_weight;

ods output solutionF=sol;
run;


data sol2;
set sol;

if _N_ = 2;
run;

data table;
set table sol2;
run;

proc print data=table;
var Effect Estimate    Lower Upper Probt;
run;





/* Fourth, introduce some revised variables based on Vermont lab variable development */





options nolabel;

data wray;
set lib.Mesasupplementaldata20180712;

idno=mesaid;

if gdpctcd3< 0 then gdpctcd3=.;
if CD14__CD16_PCTCD14<0 then CD14__CD16_PCTCD14=.;
if CD14_CD16_PCTCD14 <0 then CD14_CD16_PCTCD14=.;
if CD14DIMCD16__PCTCD14 <0 then CD14DIMCD16__PCTCD14=.;
if cd_cd25_cd127_cd6_pctcd4 < 0  then cd_cd25_cd127_cd6_pctcd4=.;

run;
```

```
proc means;
run;

proc sort data=wray;
by idno;
run;
data new;
merge e wray;
by idno;
gdpctcd3=gdpctcd3/6.1365406;
cd_cd25_cd127_cd6_pctcd42=cd_cd25_cd127_cd6_pctcd4/1.1196273;
CD14__CD16_PCTCD142 =CD14__CD16_PCTCD14 /10.2396366 ;
CD14_CD16_PCTCD142 =CD14_CD16_PCTCD14/7.0487664 ;
CD14DIMCD16__PCTCD142 =CD14DIMCD16__PCTCD14/7.5246573 ;
run;



proc means;
run;
data table;
run;



proc mixed data=new METHOD=REML EMPIRICAL;
class idno;
model sbp_adj = gdpctcd3
  age   male r2 r3 r4  cursmk
exercm1c college diabetes log_cmv bmi

 / cl ;

repeated /subject=idno;  weight samp_weight;


ods output solutionF=sol;
run;


proc mixed data=new METHOD=REML EMPIRICAL;
class idno;
model sbp_adj = cd_cd25_cd127_cd6_pctcd42
  age    male r2 r3 r4  cursmk
exercm1c college diabetes log_cmv bmi


 / cl ;
random Intercept/subject=idno;
repeated /subject=idno; weight samp_weight;

ods output solutionF=sol;
run;
```

```
proc mixed data=new METHOD=REML EMPIRICAL;
class idno;
model sbp_adj = CD14__CD16_PCTCD142

  age   male r2 r3 r4  cursmk
exercm1c college diabetes log_cmv bmi

bmi
 / cl ;
random Intercept/subject=idno;
repeated /subject=idno; weight samp_weight;

ods output solutionF=sol;
run;


proc mixed data=new METHOD=REML EMPIRICAL;
class idno;
model sbp_adj = CD14_CD16_PCTCD142

  age   male r2 r3 r4  cursmk
exercm1c college diabetes log_cmv bmi


 / cl ;
random Intercept/subject=idno;
repeated /subject=idno; weight samp_weight;

ods output solutionF=sol;
run;


proc mixed data=new METHOD=REML EMPIRICAL;
class idno;
model sbp_adj = CD14DIMCD16__PCTCD142

  age   male r2 r3 r4  cursmk
exercm1c college diabetes log_cmv bmi


 / cl ;
random Intercept/subject=idno;
repeated /subject=idno; weight samp_weight;

ods output solutionF=sol;
run;


/* This is the complete data assembly and mocdeling code for the primary results of the paper */
```
