# Supplementary material for: Natural killer cells, gamma delta T cells and classical monocytes are associated with systolic blood pressure in the multi-ethnic study of atherosclerosis (MESA)
Source: BMC Cardiovasc Disord. 2021 Jan 22;21:45. doi: 10.1186/s12872-021-01857-2 (PMC7821496; doi:10.1186/s12872-021-01857-2)
Supplement: Supplementary file 2 — Additional file 2. Supplmentary tables. [file 12872_2021_1857_MOESM2_ESM.docx]

**Table S1. Antibodies* used in the study and their catalog numbers.**

| **Assay** | **Antigen** | **Fluorophore** | **Antibody**  **(catalog number)** | **Isotype**  **(catalog number)** |
| --- | --- | --- | --- | --- |
| **CD4 Subtypes** | CD4 | Per-CP-Vio | 130-103-793 | 130-097-561 |
|  | CD45RA | APC-Vio | 130-096-604 | 130-096-822 |
|  | CD45RO | VioBlue | 130-099-044 | 130-094-671 |
|  | CD28 | PE | 130-092-921 | 130-092-212 |
|  | CD38 | PE-Vio | 130-099-151 | 130-096-638 |
|  | CD57 | APC | 130-092-141 | 130-093-176 |
|  | CD27 | VioBright FITC | 130-104-845 | 130-104-513 |
| **CD8 Subtypes** | CD8 | Per-CP-Vio | 130-097-911 | 130-097-563 |
|  | CD45RA | APC-Vio | 130-096-604 | 130-096-822 |
|  | CD45RO | VioBlue | 130-099-044 | 130-094-671 |
|  | CD28 | PE | 130-092-921 | 130-092-212 |
|  | CD38 | PE-Vio | 130-099-151 | 130-096-638 |
|  | CD57 | APC | 130-092-141 | 130-093-176 |
|  | CD27 | VioBright FITC | 130-104-845 | 130-104-513 |
| **T Cell/B Cell/NK Cell** | CD16 | Per-CP-Vio | 130-100-430 | 130-098-595 |
|  | CD19 | APC-Vio | 130-096-643 | 130-096-653 |
|  | CD3 | VioBlue | 130-094-363 | 130-094-671 |
|  | γδ TCR | PE | 130-096-869 | 130-092-212 |
|  | CD56 | PE-Vio | 130-100-676 | 130-104-616 |
|  | CD5 | APC | 130-096-577 | 130-092-214 |
|  | CD27 | VioBright FITC | 130-104-845 | 130-104-513 |
| **Monocyte Subtypes** | CD16 | Per-CP-Vio | 130-100-430 | 130-098-595 |
|  | TIE-2 | APC-Vio | 130-101-607 | 130-104-618 |
|  | CX3CR1 | PE | 130-105-837 | 130-104-612 |
|  | CD14 | PE-Vio | 130-096-628 | 130-096-638 |
|  | HLA-DR | APC | 130-095-297 | 130-091-836 |
|  | CD163 | FITC | 130-096-626 | 130-092-213 |
| **Regulatory T Cells** | CD127 | PeVio | 130-099-719 | 130-096-638 |
|  | CD6 | APC-Vio | 130-105-129 | 130-096-653 |
|  | CD25 | VioBright FITC | 130-104-274 | 130-104-575 |
|  | CD4 | APC | 130-092-374 | 130-096-653 |
| **Th1** | CD4 | APC | 130-113-222 | 130-113-434 |
|  | CD8 | FITC | 130-110-677 | 130-113-437 |
|  | IFN-γ | PE-Vio | 130-113-499 | 130-113-440 |
|  | IL-4 | PE | 130-114-842 | 130-113-438 |
|  | IL-17A | APC-Vio | 130-118-249 | 130-113-435 |

*** All Antibodies from Miltenyi Biotec**

**Systolic blood pressure interactions for subsets of primary interest**

**Interaction Testing:** These are results from a linear mixed model, using the final set of adjustment variables that go into Table 3 of the manuscript and introducing interaction terms to test whether the association between specific cell subsets is effect measure modified by sex (male), high body mass index (median split at 28.3 kg/m^2^), older age (> 65 years), or MESA race/ethnicity categories. Estimates presented are for the interaction term between immune cell subsets and potential effect measure modifier.

**Table S2: Male (Female reference)**

| **Molecular Definition** | **∆ mmHG per SD** | **95% CI Lower** | **95% CI Upper** | **p-value** |
| --- | --- | --- | --- | --- |
| CD3+γδ+ | -0.63 | -2.69 | 1.43 | 0.55 |
| CD4+CD25+CD127- | 1.10 | -1.19 | 3.39 | 0.35 |
| CD14++CD16- | 1.15 | -1.29 | 3.58 | 0.36 |
| CD14+CD16+ | -2.05 | -4.51 | 0.41 | 0.10 |
| CD14_Dim_CD16++ | 0.30 | -1.94 | 2.54 | 0.80 |
| CD3-CD56+CD16+ | 0.51 | -1.70 | 2.72 | 0.65 |
| CD4+IFN-γ+ | -0.25 | -3.27 | 2.78 | 0.87 |
| CD4+IL-17A+ | 1.55 | -0.96 | 4.07 | 0.22 |

**Table S3: Body Mass Index (>28.3 kg/m^2^)**

| **Molecular Definition** | **∆ mmHG per SD** | **95% CI Lower** | **95% CI Upper** | **p-value** |
| --- | --- | --- | --- | --- |
| CD3+γδ+ | 1.60 | -0.82 | 4.02 | 0.20 |
| CD4+CD25+CD127- | 0.16 | -1.16 | 1.49 | 0.81 |
| CD14++CD16- | 0.38 | 0.05 | 0.71 | 0.03 |
| CD14+CD16+ | 0.54 | -0.46 | 1.54 | 0.29 |
| CD14_Dim_CD16++ | 1.13 | -0.04 | 2.29 | 0.06 |
| CD3-CD56+CD16+ | 1.52 | 0.19 | 2.84 | 0.03 |
| CD4+IFN-γ+ | -0.37 | -1.95 | 1.20 | 0.64 |
| CD4+IL-17A+ | 0.77 | -0.98 | 2.51 | 0.39 |

**Table S4: Age> 65 years**

| **Molecular Definition** | **∆ mmHG per SD** | **95% CI Lower** | **95% CI Upper** | **p-value** |
| --- | --- | --- | --- | --- |
| CD3+γδ+ | 0.59 | -1.47 | 2.65 | 0.57 |
| CD4+CD25+CD127- | -0.42 | -1.72 | 0.88 | 0.53 |
| CD14++CD16- | -0.02 | -0.40 | 0.35 | 0.90 |
| CD14+CD16+ | 0.22 | -0.78 | 1.22 | 0.66 |
| CD14_Dim_CD16++ | -0.13 | -2.71 | 2.45 | 0.92 |
| CD3-CD56+CD16+ | 0.40 | -1.51 | 2.31 | 0.68 |
| CD4+IFN-γ+ | 0.24 | -1.75 | 2.24 | 0.81 |
| CD4+IL-17A+ | 0.28 | -1.54 | 2.10 | 0.77 |

**Table S5: Race interactions (White is reference)**

| **Molecular Definition** | **∆ mmHG per SD** | **95% CI Lower** | **95% CI Upper** | **p-value** |
| --- | --- | --- | --- | --- |
| CD3+γδ+ (Asian) | -1.51 | -3.73 | 0.72 | 0.18 |
| CD3+γδ+ (Black) | 1.52 | -1.48 | 4.52 | 0.32 |
| CD3+γδ+ (Hispanic) | -0.95 | -4.14 | 2.23 | 0.56 |
| CD4+CD25+CD127- (Asian) | 1.38 | -2.40 | 5.15 | 0.47 |
| CD4+CD25+CD127- (Black) | -0.83 | -3.60 | 1.95 | 0.56 |
| CD4+CD25+CD127- (Hispanic) | 0.24 | -2.84 | 3.31 | 0.88 |
| CD14++CD16- (Asian) | 1.85 | -2.35 | 6.05 | 0.39 |
| CD14++CD16- (Black) | 0.68 | -2.43 | 3.78 | 0.67 |
| CD14++CD16- (Hispanic) | 0.35 | -3.15 | 3.85 | 0.85 |
| CD14+CD16+ (Asian) | -0.22 | -4.08 | 3.63 | 0.91 |
| CD14+CD16+ (Black) | -0.03 | -3.27 | 3.20 | 0.98 |
| CD14+CD16+ (Hispanic) | -0.58 | -3.87 | 2.71 | 0.73 |
| CD14_Dim_CD16++ (Asian) | -2.99 | -8.65 | 2.67 | 0.30 |
| CD14_Dim_CD16++ (Black) | -1.21 | -4.18 | 1.76 | 0.42 |
| CD14_Dim_CD16++ (Hispanic) | 0.21 | -3.25 | 3.67 | 0.91 |
| CD3-CD56+CD16+ (Asian) | 0.44 | -2.58 | 3.47 | 0.77 |
| CD3-CD56+CD16+ (Black) | 0.25 | -2.65 | 3.16 | 0.86 |
| CD3-CD56+CD16+ (Hispanic) | 2.24 | -0.51 | 4.99 | 0.11 |
| CD4+IFN-γ+(Asian) | -1.83 | -7.18 | 3.52 | 0.50 |
| CD4+IFN-γ+(Black) | -0.59 | -4.16 | 2.98 | 0.75 |
| CD4+IFN-γ+(Hispanic) | 2.75 | -1.43 | 6.94 | 0.20 |
| CD4+IL-17A+(Asian) | -2.82 | -6.47 | 0.84 | 0.13 |
| CD4+IL-17A+(Black) | 1.36 | -3.09 | 5.81 | 0.55 |
| CD4+IL-17A+(Hispanic) | -2.10 | -5.61 | 1.41 | 0.24 |
