# Supplementary material for: Natural killer cells, gamma delta T cells and classical monocytes are associated with systolic blood pressure in the multi-ethnic study of atherosclerosis (MESA)
Source: BMC Cardiovasc Disord. 2021 Jan 22;21:45. doi: 10.1186/s12872-021-01857-2 (PMC7821496; doi:10.1186/s12872-021-01857-2)
Supplement: Supplementary file 3 — Additional file 3. Sample MESA informed consent form. [file 12872_2021_1857_MOESM3_ESM.pdf]

Form C (Revised 01/2001) (WORD)

CLINICAL INVESTIGATION CONSENT FORM

The Johns Hopkins Medical Institutions
(The Johns Hopkins Hospital
The Johns Hopkins Bayview Medical Center, etc.)

Date/Revision: **May 3, 2001** APPLICATION NO: **99-11-10-06**

Title of Research Project:

Multi-Ethnic Study of Atherosclerosis (MESA)

Patient I.D. Plate

Explanation of Research Project to Subject

Title of Research Project: Multi-Ethnic Study of Atherosclerosis (MESA)

PURPOSE OF STUDY

The purpose of MESA is to study heart disease and diseases of the blood vessels in the early stages to determine why some people develop more serious conditions such as heart attack and stroke. The MESA study will attempt to learn if the early stages of heart disease (while a person still feels healthy) can be identified, and also to learn why this condition becomes more severe in some people. In order to learn this information, a group of 1087 people living in Baltimore will be followed for several years. You qualify as a participant because you have been selected from among people living near the Baltimore subway line connecting with the Johns Hopkins Hospital, where our study is based.

PROCEDURES

If you agree to participate in the MESA study, you will be asked to come to four examinations over the next six years (about one visit every 1 and a half years). The first visit will take approximately 6 hours. The other visits, which will be spread out over six years, will take approximately 4 hours each. The standard procedures (see below) will be conducted at the field center clinic. The special procedures will be done at the Johns Hopkins Hospital.

Standard procedures:

At each visit you will receive a physical exam in which your blood pressure, height, weight, and waist will be measured. You will also be asked questions about your medical history, physical health, use of tobacco and alcohol, your diet and exercise habits, and stressful things about your life which may be important to your health. Approximately 75 ml of your blood (about 5 tablespoons) will be drawn and analyzed for cholesterol and other fats in the blood, blood sugar, and other substances.

Special procedures:

a) Computed tomography (CT): As part of the MESA Study, you will receive an ultrafast CT scan (a new type of X-ray) at the Johns Hopkins Hospital. This test is only done to measure the amount of calcium in the arteries of your heart. For this test, you will lie on a table and be moved through a large (approximately four feet in diameter) donut shaped x-ray machine. During the scanning process, your chest is in the donut, but your head is free. The scan will be done twice in order to increase the accuracy. The CT test should take no more than 20 minutes. Before having the CT scan, women will be asked if they might be pregnant, and if they are less than one year from the time of their last menstrual period, a rapid pregnancy test will be performed. Although the levels of radiation used in the study are within Federal guidelines for research of this type, radiation risk builds up over a lifetime, so any additional exposure should be carefully considered.

THIS CONSENT FORM CONTINUES ON THE NEXT PAGE

b) **Magnetic resonance imaging (MRI)**:The MRI exam is limited to the evaluation of the size and function of your heart. No other organs or systems will be evaluated as a part of this study. You will be asked to lie on your back in a machine for a period of 30 minutes while the imaging takes place. MRI is a non-invasive procedure that will take pictures by using magnetic fields. MRI does not use ionizing radiation (such as x-rays). During that time you will be asked to wear earplugs or earphones to avoid the high noise levels that are produced by the equipment.

c) **Ultrasound**: An ultrasound test will be done on the arteries in your neck to measure their size and function. This test is painless, involves no known risk and takes about 20 minutes. You will lie on a table while a small hand-held ultrasound probe is placed lightly on the right and left side of your neck.

d) **Endothelial function**: An endothelial function test (blood vessel function test) will be done to determine how the arteries in your arm respond to increased blood flow. After a 15 minute rest period, a blood pressure cuff will be placed on your arm and inflated several times while recordings are made.

e) **Genetic/DNA testing**: You will be asked to allow genetic testing on the blood samples that will be collected and stored as part of the MESA study. Your blood samples may be used to prepare DNA or cell lines. Cell lines are blood cells that have been treated so they will live for long periods of time. Genetic testing is necessary to study genes important to the development of heart and vascular disease. The DNA will be stored in a central site.

f) **Follow-up:** You will be contacted by phone or mail once a year and asked about your health over the past year. If you are hospitalized or admitted to a convalescent or nursing home, MESA staff will review your hospital or convalescent/nursing home records to determine the reason for your admission and verify the diagnosis. We would also want to be able to access information about cause of death, if anything unfortunate should happen within the 10 years of the study. MESA staff may also contact your physician or a person you indicate could answer questions for you, if you are unable to answer for yourself.

RISKS AND DISCOMFORTS

Risks:

The procedures to be used in this study are considered to be safe. The risks associated with the clinic exam are minimal. Risks associated with the drawing of a blood sample are discomfort at the site of needle insertion, bruising (black and blue discoloration) at the site, and rarely, faintness.

You should be aware that insurance companies sometimes use information on genetic testing to deny coverage to applicants. This study involves research in genetic testing aimed at developing such testing in the future. Therefore, the information obtained in this research cannot provide any meaningful genetic information about participants. Since this is the case, if you are asked, you should state that you have not had a genetic test.

The radiation exposure you will receive from the CT scan is equivalent to an exposure of 0.3 rems to your whole body. Naturally occurring radiation (cosmic radiation, radon, etc.) produces whole body radiation exposures of about 0.3 rem per year. Occupationally exposed individuals are permitted to receive whole body exposures of 5 rems per year. You will receive two scans at the first visit. Some people may get two scans in visit 2 and two in visit 3, for a total of 6 scans. A smaller proportion of the study participants will receive 2 additional scans in visit 4.

The MRI machine does not use ionizing radiation (such as x-rays) and is thought, at this time, to pose no significant risk. You may need to wear earplugs or earphones since operation of the machine can produce high noise levels which may be uncomfortable. Some people may experience psychological discomfort in the scanner if they are uncomfortable in tight places (known as claustrophobia). If you become anxious, you may tell the technician and you will be removed from the machine.

THIS CONSENT FORM CONTINUES ON THE NEXT PAGE

BENEFITS

Benefits:

One benefit of participating in this study is that a free evaluation of your health will be performed. Information from the tests will be available to you and to your doctor if you so desire. If a health condition is detected during this evaluation, your doctor will be notified, if you authorize the study staff to do so. However, the MESA study is not intended to provide medical care or interfere with your relationship with your own doctor. You will be referred to your own doctor for follow-up of all medical information obtained by the study. If you do not have a local doctor, you can be referred to one, if you so desire. The information learned from this study will increase scientific knowledge about the causes of early heart disease and diseases of the blood vessels.

COSTS AND PAYMENTS

There are no costs associated with participating in this study. You will be reimbursed for your mileage or transportation. We will provide transportation between the study clinic and the Johns Hopkins Hospital.

QUESTIONS YOU MAY HAVE ABOUT THE RESEARCH STUDY

This consent form explains the research study. Please read it carefully. Ask questions about anything you do not understand. If you do not have questions now, you may ask later. During the study, you will be told any new facts that could affect whether you want to stay in the study. If the study relates to a health problem you have, we will explain what other treatment could be given outside the research. You should understand those options before you sign this form. If you have questions you should call the principal investigator, Dr. Moyses Szklo, at (410) 955-3462, or the Project Director, Mrs. Joel Hill, at (410) 944-6780.

PRIVACY INFORMATION:

We will keep the study information private to the extent possible by law. However, State law requires us to report certain contagious diseases or if we find information about child abuse. Also, under certain conditions, people responsible for making sure that the research is done properly may review your study records. This might include people from Johns Hopkins, the National Institutes of Health, the Food and Drug Administration, or the sponsoring company (if any). All of these people are also required to keep your identity confidential. Otherwise, the information that identifies you will not be given out to people who are not working on the study, unless you give permission.

You have the option of having your examination results released to your personal physician. Doing so would allow him/her to further evaluate or treat any medical condition(s) we might uncover. However, identification of new medical conditions could affect your ability to get health insurance in the future or the cost of such future health insurance.
Your personal identity will not be revealed in any publications or release of results. The information obtained, however, may be used for statistical or scientific purposes with your right of privacy retained. Study records may be kept indefinitely for analysis and follow-up.

Results from genetics testing will not be released, placed in your medical records or shared in any way with your relatives, personal physician, insurance companies, or any other third party unless you authorize MESA staff, in writing, to do so. Genetic information has been specifically exempted from the Freedom of Information Act regulations and is not accessible to the American public.

THIS CONSENT FORM CONTINUES ON THE NEXT PAGE

DNA will be provided only to qualified researchers (we have an extensive screening process) without any identifying information, and only if the research is directly related to the goals of the MESA study. These investigators will therefore not know who you are. Each researcher and staff member will be required to sign a legal binding Confidentiality Agreement stating that they 1) won't ever try to identify you, 2) won't try to re-contact you based on the results, 3) won't release any research information to any relatives or third parties, 4) won't redistribute DNA or analysis results to other researchers or third parties, 5) will make every effort to avoid inadvertent disclosure of any kind, 6) will use the DNA analysis results only for specific agreed-upon purposes, 7) will proceed at all times through the established MESA standard rules and regulations.

To help insure your privacy, a Certificate of Confidentiality has been obtained from the National Heart, Lung and Blood Institute for this study. This Certificate means that researchers cannot be forced to tell people who are not connected with the study, including courts, about your participation, without your written consent. However, if the researchers learn that you or someone else is in serious danger or harm, they would make disclosures, if necessary, to protect you and other people.

IF CASE OF INJURY:

If you are injured as a result of being in the study, or think you have not been treated fairly, please contact Dr. Moyses Szklo, at (410)-955-3462. The services at the Johns Hopkins Hospital or the Johns Hopkins Bayview Medical Center will be open to you in case of any such injury. However, the Johns Hopkins University, the Johns Hopkins Hospital, the Johns Hopkins Bayview Medical Center, and the Federal Government do not have a program to pay you if you are hurt or have other bad results which are not the fault of the study doctors.

You and your insurance company will be responsible for payment of any treatment or hospitalization you require if you are injured as a result of being in the study. It is up to you to check with your insurance company before you start this study to find out what your insurance company would pay for.

QUESTIONS ABOUT YOUR RIGHTS AS A RESEARCH SUBJECT:

If you have any questions about your rights as a subject in a research project, you should call the Joint Committee on Clinical Investigation at (410) 955-3008, or the Johns Hopkins Bayview Medical Center Institutional Review Board for Human Research (410) 550-1853 to receive help or advice.

JOINING OF YOUR OWN FREE WILL (Volunteering for the study)

You do not have to join this or any research study. If you do join, and later change your mind, you may quit at any time. If you refuse to join the study, you will not be penalized or lose any benefits to which you are otherwise entitled.

WHAT YOUR SIGNATURE MEANS:

Your signature below means that you understand the information given to you about the study and in this consent form. If you sign the form it means that you agree to join the study.

WE WILL GIVE YOU A COPY OF THIS CONSENT FORM

THIS CONSENT FORM CONTINUES ON THE NEXT PAGE

STUDY APPROVED FOR ENROLLMENT OF: __ Adults Only __ Adults and Children __ Children only

NOT VALID WITHOUT THE COMMITTEE OR IRB STAMP OF CERTIFICATION

APPROVED

MAY 0 4 2001

JOINT COMMITTEE ON CLINICAL INVESTIGATION

PROTOCOL WILL EXPIRE: 2/27/02

[redacted]

RPN NO 99-11-10-06

Form C (Revised 01/2001)

Subject's signature (including children, when applicable) **Date**

Signature of Parent or Legal Guardian (when applicable) **Date**

Surrogate Signature for Subjects not Competent to Give Consent **Date**

Relationship of Surrogate to Subject: ____________________

Signature of Investigator or IRB/JCCI Approved Designee **Date**

Witness to Consent Procedures(Optional unless subject is illiterate, or unable to sign) Date

NOTE: A COPY OF THE SIGNED CONSENT FORM MUST BE KEPT BY THE PRINCIPAL INVESTIGATOR AND A COPY OF THE CONSENT FORM MUST BE PLACED IN THE PATIENT'S RECORD

THIS CONSENT FORM CONTINUES ON THE NEXT PAGE

CONSENT TO RELEASE INFORMATION TO MY HEALTH CARE PROVIDERS

I authorize the Multi-Ethnic Study of Atherosclerosis (MESA) to release the findings from tests and examinations to my physician.

Name of physician: ____

MEDICAL RECORDS RELEASE

I do ___ do not ___ authorize the Multi-Ethnic Study of Atherosclerosis (MESA) to obtain medical records from my physician, or from any hospitals or convalescent/nursing homes where I might be admitted, death certificates and coroner's reports from the appropriate city or state agencies, and information from state and other cancer surveillance systems

______________________________ ______________________________
Signature of Participant **Date**

______________________________ ______________________________
Person obtaining consent **Date**

NOTE: A COPY OF THE SIGNED CONSENT FORM MUST BE KEPT BY THE PRINCIPAL INVESTIGATOR AND A COPY OF THE CONSENT FORM MUST BE PLACED IN THE PATIENT'S RECORD

THIS CONSENT FORM CONTINUES ON THE NEXT PAGE

CONSENT FOR GENETICS TESTING

I give my permission to:	Yes	No
Prepare DNA from my blood samples	☐	☐
Create a cell line from my blood cells.	☐	☐
Test my DNA for genes related to the main goals of the study: heart and vascular diseases	☐	☐
Test my DNA for genes related to the secondary goals of the study: other health conditions	☐	☐
Allow researchers from private companies who wish to develop diagnostic lab tests or pharmaceutical therapies that could benefit many people to have access to my DNA (Note: you or your heirs will not benefit financially from this, nor will your cell line or DNA be sold to anyone)<br><br>Note: Neither your name nor other identifying information would be given to these researchers.	☐	☐

________________________ ________________________

Signature of Participant **Date**

________________________ ________________________

Person obtaining consent **Date**

NOTE: A COPY OF THE SIGNED CONSENT FORM MUST BE KEPT BY THE PRINCIPAL INVESTIGATOR AND A COPY OF THE CONSENT FORM MUST BE PLACED IN THE PATIENT'S RECORD
